# Correction: Similarities and differences in spatial and non-spatial cognitive maps

**DOI:** 10.1371/journal.pcbi.1008384

**Published:** 2020-10-21

**Authors:** Charley M. Wu, Eric Schulz, Mona M. Garvert, Björn Meder, Nicolas W. Schuck

The funding statement for this article should read as follows:

“NWS was funded by an Independent Max Planck Research Group grant awarded by the Max Planck Society and a Starting Grant from the European Union (ERC-2019-StG-REPLAY- 852669). ES is supported by the Max Planck Society and the Jacobs Foundation. The funders had no role in study design, data collection and analysis, decision to publish, or preparation of the manuscript.”
