# Supplementary figures and images for: Similarities and differences in spatial and non-spatial cognitive maps
Source: PLoS Comput Biol. 2020 Sep 9;16(9):e1008149. doi: 10.1371/journal.pcbi.1008149 (PMC7480875; doi:10.1371/journal.pcbi.1008149)

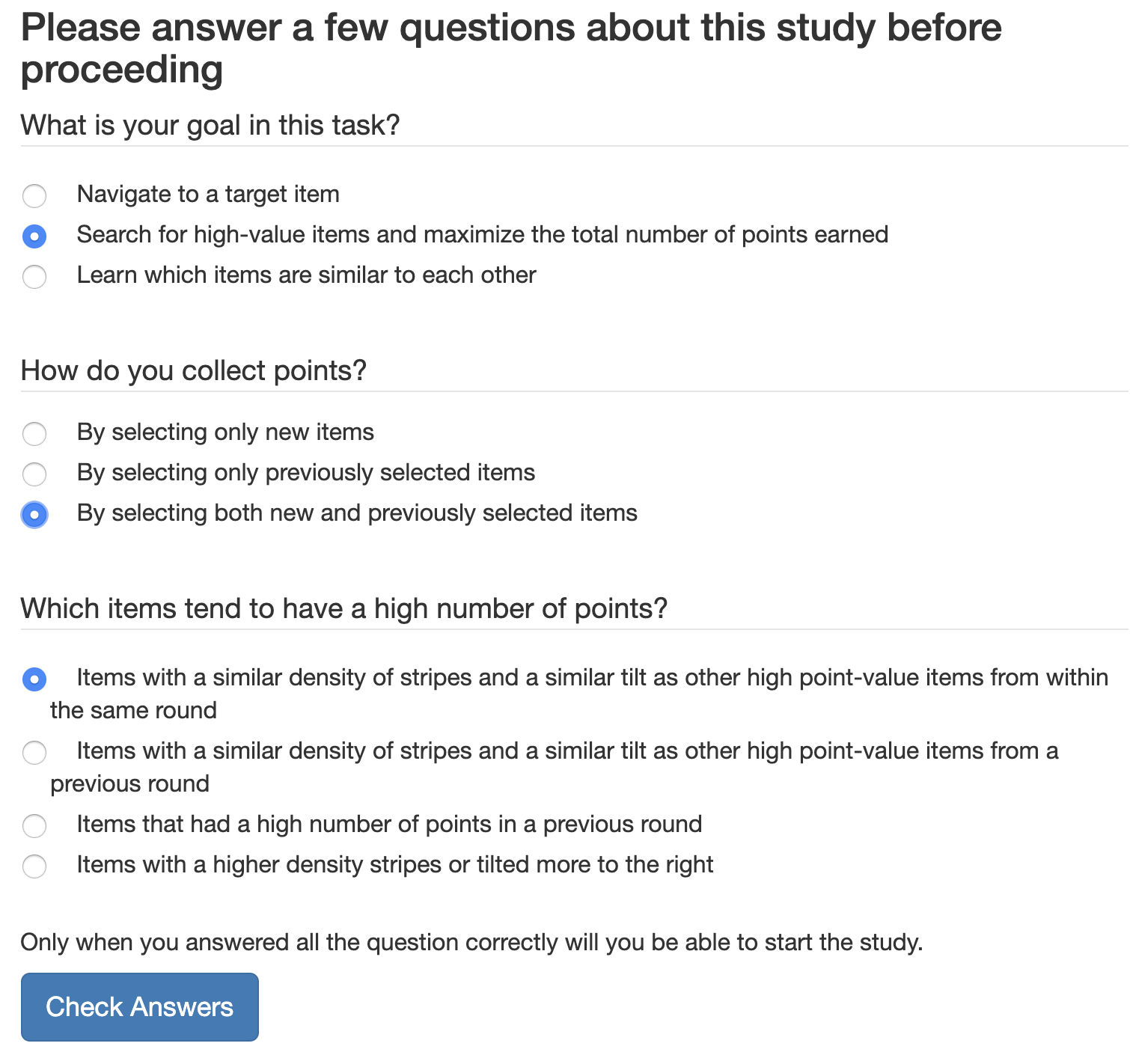

Supplement: S11 Fig — The correct answers are highlighted. (TIFF) [file pcbi.1008149.s011.tiff]

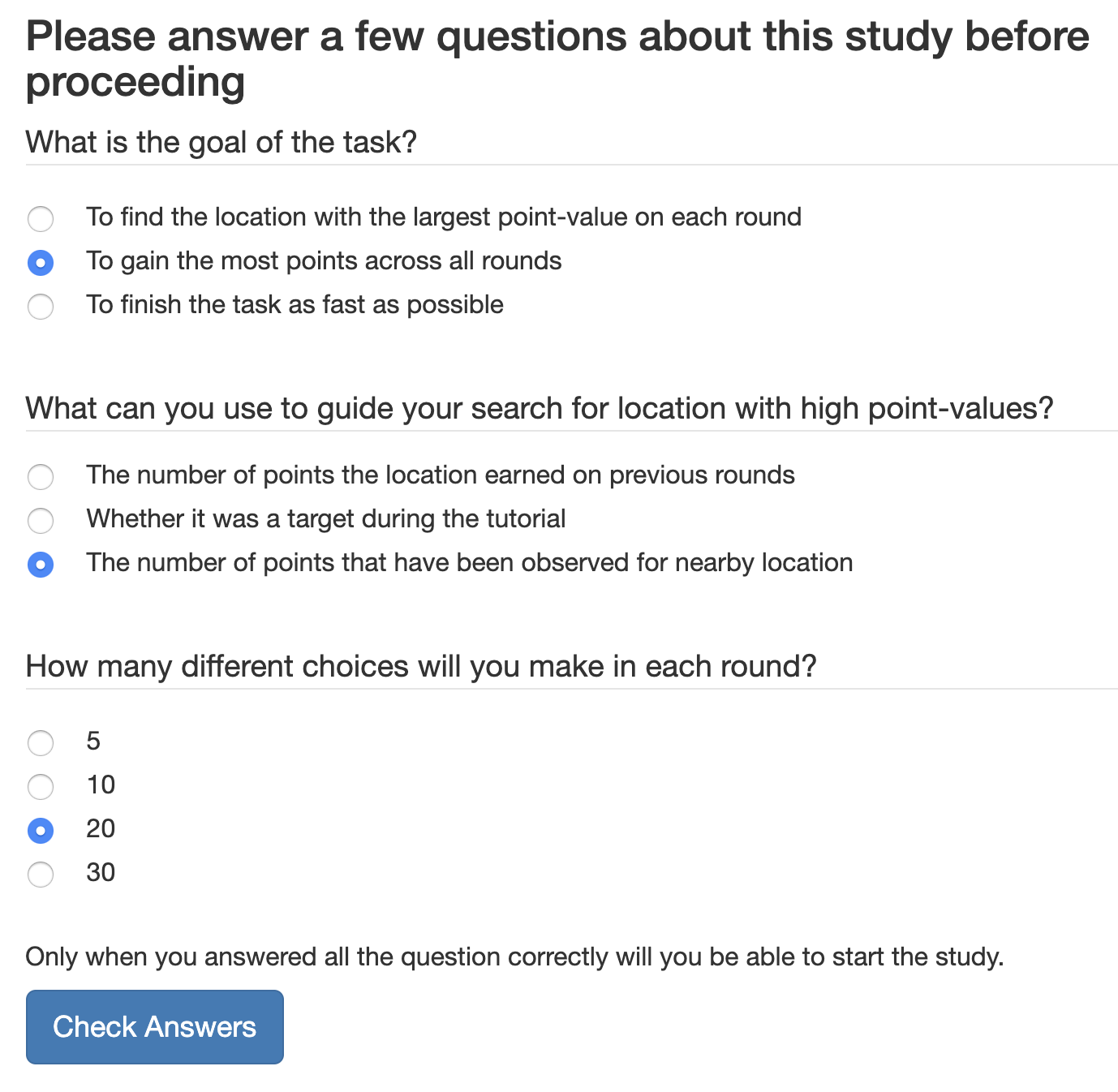

Supplement: S12 Fig — The correct answers are highlighted. (TIFF) [file pcbi.1008149.s012.tiff]
