# Supplementary material for: Similarities and differences in spatial and non-spatial cognitive maps
Source: PLoS Comput Biol. 2020 Sep 9;16(9):e1008149. doi: 10.1371/journal.pcbi.1008149 (PMC7480875; doi:10.1371/journal.pcbi.1008149)
Supplement: S1 Table — (PDF) [file pcbi.1008149.s013.pdf]

	Distance Between Choices		Distance from Initial Position	
Predictors	*Est.*	*95% HPD*	*Est.*	*95% HPD*
Intercept	7.04	6.77 – 7.31	4.21	4.00 – 4.41
PreviousReward	-0.06	-0.06 – -0.06	0.01	0.01 – 0.01
Spatialtask	1.03	0.68 – 1.38	-0.2043	-0.67 – -0.18
PreviousReward:Spatialtask	-0.01	-0.02 – -0.01	0.01	0.004 – 0.01
**Random Effects**				
σ^2	1.10		1.08	
τ_{00}	7.22		8.34	
N	129		129	
Observations	44118		44118	
Bayesian R^2	.539		.118	

Note: We report the posterior median (Est.) and 95% highest posterior density (HPD) interval. σ^2 indicates the individual-level variance and τ_{00} indicates the variation between individual intercepts and the average intercept. See Methods for full specification of model structure and priors.
