# Supplementary material for: Similarities and differences in spatial and non-spatial cognitive maps
Source: PLoS Comput Biol. 2020 Sep 9;16(9):e1008149. doi: 10.1371/journal.pcbi.1008149 (PMC7480875; doi:10.1371/journal.pcbi.1008149)
Supplement: S2 Table — (PDF) [file pcbi.1008149.s014.pdf]

	Model Prediction		Model Uncertainty	
Predictors	*Est.*	*95% HPD*	*Est.*	*95% HPD*
Intercept	12.42	10.45 – 14.34	0.92	0.86 – 0.98
ParticipantJudgment	0.82	0.75 – 0.89	-0.02	-0.02 – -0.01
Spatialtask	1.34	-0.33 – 3.04	0.002	-0.08 – 0.08
ParticipantJudgment:Spatialtask	0.03	-0.04 – 0.11	-0.01	-0.02 – 0.001
**Random Effects**				
σ^2	183.49		0.06	
τ_{00}	346.35		0.03	
N	129		129	
Observations	2580		2580	
Bayesian R^2	.437		.674	

Note: We report the posterior median (Est.) and 95% highest posterior density (HPD) interval. In the first model (Model Prediction), participant judgments in the range [1,100] are used to predict the GP posterior mean, whereas the second model (Model Uncertainty) uses confidence judgments in the range [1,11] to predict the GP posterior variance. All GP posteriors are computed based on individual participant λ-values, estimated from the corresponding bandit task. σ^2 indicates the individual-level variance and τ_{00} indicates the variation between individual intercepts and the average intercept. See Methods for full specification of model structure and priors.
