# Supplementary material for: Similarities and differences in spatial and non-spatial cognitive maps
Source: PLoS Comput Biol. 2020 Sep 9;16(9):e1008149. doi: 10.1371/journal.pcbi.1008149 (PMC7480875; doi:10.1371/journal.pcbi.1008149)
Supplement: Attachment — Submitted filename: Neurogrid_resubmission_letter.pdf [file pcbi.1008149.s015.pdf]

Similarities and differences in spatial and non-spatial cognitive maps
PLOS Computational Biology response letter

Charley M. Wu, Eric Schulz, Mona M. Garvert, Björn Meder and Nicolas W. Schuck

May 1, 2020

Dear Dr. Marinazzo,

We are grateful for the constructive feedback we received and for the opportunity to submit a revision. In revising our manuscript, we have aimed to address your critiques about statistics and reporting of data, and all the further issues discussed by the reviewers.

We greatly appreciate your effort and the effort of the reviewers and hope that our revised manuscript meets the standards of *PLOS Computational Biology*. We address yours and the reviewers' comments individually below. Thank you very much, and we are looking forward to hearing from you.

Best regards,

Charley M. Wu, Eric Schulz, Mona M. Garvert, Björn Meder and Nicolas W. Schuck

Contents

1 Editor **3**
1.1 General remarks . . . 3
1.2 Plot all data points . . . 3
1.3 Robust statistics . . . 4
1.4 Linear fits . . . 5

2 Reviewer 1 **7**
2.1 Generalizability of results . . . 7
2.2 Stimuli . . . 7
2.3 Other models . . . 9
2.4 Claims . . . 10
2.5 Code . . . 11
2.6 Typo . . . 12

3 Reviewer 2 **13**
3.1 General remarks . . . 13
3.2 Clarification on the training task . . . 13
3.3 Distance dependent generalization . . . 14
3.4 Scope of behavioral analyses explained by the GP . . . 15
3.5 Differences in GP learning curves across environments . . . 16
3.6 Differences in exploration parameter . . . 17

4 Reviewer 3 **20**
4.1 General remarks . . . 20
4.2 Differences between environments . . . 20
4.3 Attention to feature dimensions and transfer . . . 20
4.4 Model comparison . . . 25
4.5 Mismatch in reward structure . . . 25
4.6 Other models and theoretical ideas . . . 26
4.7 Suggestion for abstract . . . 28
4.8 Suggestions for introduction . . . 29
4.9 Figure suggestion . . . 30

1 Editor

1.1 General remarks

> The paper was overall well received, but some important issues need to be addressed, in particular involving better motivating your approach and situating it in the state of the art, together with commenting on its generalizability.

We are grateful for the constructive feedback and for the opportunity to submit a revision. The revised manuscript has been greatly improved based on the reviewer feedback. These changes include new and insightful analyses, more rigorous statistical evidence supporting our main findings, and a more comprehensive integration of different theoretical ideas in the field.

1.2 Plot all data points

> please always report all the data points, as you do in most figures, instead of bar plots with confidence bars

This is a helpful suggestion for increasing the transparency of the paper. One data figure that did not include all data points was Fig. 2c, since it provided a different presentation of the same data shown in Fig. 2a in order to highlight the order effect. However, to avoid any potential confusion, we have replaced the bar graphs with a plot showing the full data (Fig. R1 below). We have also corrected a minor error with the labeling of the Bayes factor comparisons, and changed the ordering of the task order factors for consistency with other plots in the paper.

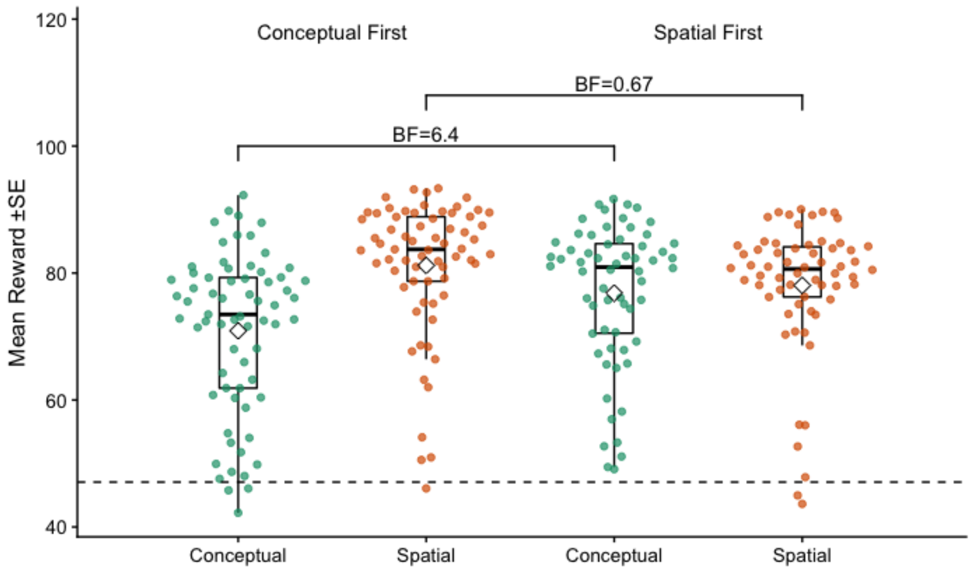

Figure R1: Task order effect. Reproduced from Figure 2c.

The revised manuscript also contains two barplots depicting results from simulations (Fig 3b and 3d). In this case, either only a single data point was available (exceedance probability; Fig. 3b), or the bars depict the mean across 10k simulations, where variability is negligible.

1.3 Robust statistics

> removing outliers messes with the degrees of freedom. Several alternative approaches exist, excellent robust alternatives are proposed in this paper Wilcox, R. R., & Rousselet, G. A. (2018). A Guide to Robust Statistical Methods in Neuroscience. Current Protocols in Neuroscience, 82(1). doi:10.1002/cpns.41 (open access here `https://www.biorxiv.org/content/10.1101/151811v1`). The same paper also suggests multivariate robust linear regression as an alternative to ANOVA

We thank the Editor for this paper recommendation. Robust and proper use of statistics is a very important consideration in our paper, and we appreciate suggestions for improving our level of rigor. Below, we highlight the key changes we have made, based on incorporating best practices from both frequentist and Bayesian statistical frameworks.

Outliers

All primary analyses involve the full data without removing any outliers. The only case where we removed outliers was for a secondary comparison of model parameters, where we used a combination of both rank-based analyses (without any omitted data) and standard t-tests and Pearson correlations after applying a conservative outlier removal procedure. We relied on a combination of both primary and secondary analyses to ensure a robust interpretation of our results, where we reasoned that robust effects would be found in both analyses.

Our primary comparison used the Mann-Whitney-U test for independent samples, or the Wilcoxon signed-rank test for paired samples to compare parameter estimates based on the medians of the data. This form of robust analysis is consistent with the recommendations proposed by Wilcox and Rousselet (2018). In addition, we used Kendall's τ for testing rank correlations of parameter estimates, also consistent with the recommendations of Wilcox and Rousselet (2018).

For extra precaution that outliers did not influence our results, we performed additional analyses using a conservative outlier removal procedure based on Tukey's fences (removing values larger than Q3 + $1.5\times$IQR) combined with standard t-tests and Pearson's correlations.

This is where our approach differed from Wilcox and Rousselet (2018), which recommends using trimmed or Winsorized means for performing outlier removal. However, their approach alters the degrees of freedom even more aggressively than our approach (e.g., a 20% trimmed mean reduces the effective sample size by 40%), while also lacking a Bayesian implementation for multivariate data (i.e., paired comparisons and correlations), which is our primary focus here.

In light of the issue you raised about outliers interfering with the degrees of freedom, we have decided the best approach will be to omit our secondary analyses involving outlier removal and instead rely on Mann-Whitney-U and Wilcoxon signed-rank tests for comparisons, and Kendall's τ for rank correlations. These approaches are robust to outliers (Wilcox & Rousselet, 2018) and do not alter the degrees of freedom of the original data. Ultimately, the interpretation of our main results remain unaltered.

ANOVA

We have replicated all ANOVA results using multivariate robust linear regression as specified by Wilcox and Rousselet (2018), using a 20% trimmed means method. For readability, we have kept the standard ANOVA results in the main text, while presenting the multivariate robust linear regression variant in the interactive code notebooks published in the online supplement (`https://github.com/charleywu/cognitivemaps`). We provide a comparison of the two methods below, which reveal the same pattern of results:

Analysis		Two-way mixed ANOVA Df	F value	Pr(>F)	Robust Two-Way Mixed ANOVA Using 20% Trimmed Means Df	F value	Pr(>F)
Reward							
	environment	1, 127	9.4	.003	1, 71.8	22.0	<.001
	task	1,127	35.8	<.001	1, 71.5	36.2	<.001
	environment:task	1, 127	1.7	.197	1, 71.5	1.4	.246
Distance							
	environment	1, 127	0.1	.727	1,70.4	0.6	.459
	task	1, 127	13.8	<.001	1, 69.0	4.7	.034
	environment:task	1, 127	1.3	.254	1, 69.0	0.9	.347

Regression

For additional statistical rigor, we have added a new section to the Methods specifying the priors and random effects structure of our Bayesian mixed effects model. In doing so, we have revised and re-run all analyses using the priors recommended by Gelman and Hill (2006) and random effects structure recommended by Barr, Levy, Scheepers, and Tily (2013). All results have been replicated.

> **Mixed Effects Regression**
>
> Mixed effects regressions are performed in a Bayesian framework with `brms` (Bürkner, 2017) using MCMC methods (No-U-Turn sampling (Hoffman & Gelman, 2014) with the proposal acceptance probability set to .99). In all models, we use a maximal random effects structure (Barr et al., 2013), and treat participants as a random intercept. Following Gelman and Hill (2006) we use the following generic weakly informative priors:
>
> $$b_0 \sim \mathcal{N}(0,1) \quad (1)$$
> $$b_i \sim \mathcal{N}(0,1) \quad (2)$$
> $$\sigma \sim \text{Half-}\mathcal{N}(0,1) \quad (3)$$
>
> All models were estimated over four chains of 4000 iterations, with a burn-in period of 1000 samples.

We have also removed the intra-class correlations (ICC) from the regression tables, since the mixed design of the experiment violates the underlying assumptions behind how it is computed under a Bayesian regression framework (Bobak, Barr, & O'Malley, 2018).

1.4 Linear fits

> some data appear to be distributed in a very non-linear way, questioning the linear fits

The learning curves shown in Figure 2d, the relation between previous reward and distance in Fig. 2f and Fig. S4c, indeed seem nonlinear. In these cases, however, our hypotheses were merely directional,

reflecting ideas about increases over time or other directed relations. We had no specific prediction about the shape of the relationship (e.g., linear or exponential). Therefore, as this was not one of our main results, we did not think it was appropriate to introduce added complexity by using a non-linear regression model. To clarify this, we have changed figure captions to point out the non-linearity where appropriate and have added an additional rank correlation test to the caption for Figure S4c:

> Distance from the random initial starting point in each trial as a function of the previous reward value. Each dot is the aggregate mean, while the lines show the fixed effects of a Bayesian mixed-effects model (see Table S1), with the ribbons indicating the 95% CI. *The relationship is not quite linear, but is also found using a rank correlation* ($r_\tau = .18$, $p < .001$, $BF > 100$). The dashed line indicates random chance. [emphasis added]

2 Reviewer 1

2.1 Generalizability of results

> Overall, I am unsure about how generalisable these results are, given the authors have not sufficiently proposed any strong theoretical constraints on their hypotheses. I would have loved to have seen a higher-level theoretical account as to why they designed their experiment the way they did, why they suspected sampling strategies would differ (or perhaps they did not?), and especially why they chose the stimuli used. I will elaborate below what I mean and how I think some of these issues can be addressed.

We thank the reviewer for addressing important high-level theoretical implications of our paper. We respond to these comments below. We believe that addressing these issues has substantially improved the quality of our paper.

2.2 Stimuli

> Firstly, in terms of the stimuli: I believe that Gabor patches are not confirmed to be homogeneously mappable to 2D. This might indicate that while participants can achieve high accuracy in the training task (which was matching a stimulus to target stimulus by moving in 2D space) the higher cognitive demands of the test phase (which involves exploration as well) would impair participants' accuracy if Gabor patches are harder to map onto 2D. In other words, due to the nature of the stimuli the test phase could be harder for Gabor patches. To assuage my worries, the authors could show that the stimuli in the two cases (spatial vs conceptual) are indeed 2D and indeed homogeneously distributed in their respective domains. What I mean is that Gabor patches might not all be equally easy to tell apart as a function of their frequency and orientation. And therefore, arguably, the spatial case could be seen as more homogenous. One way to explore this is to first ascertain if the stimuli are 2D — something like Ahlheim and Love (2018, the code is open source) could be run on the raw pixels of what the participants see to ensure both sets of stimuli have the same dimensionality. Alternatively, other methods of investigating this are possible. After that, assuming that both spaces are found to be (roughly) 2D, the issue of the homogeneity of the spaces can be addressed (thank you to Sebastian Bobadilla Suarez for input on this issue). Is moving e.g., one step in 2D frequency/orientation space also one step in the stimulus space of the Gabor stimuli? Mutatis mutandis for the spatial case, of course, which I suspect is 2D and homogenous.

We thank Reviewer 1 for the detailed feedback on our paper, and for taking the time to discuss our paper with other knowledgeable scientists in the field. These concerns are closely related to the central goal of our experiment, which was to develop a balanced and appropriate task for testing the behavioral implications of domain differences in spatial versus non-spatial environments. In other words: the purpose of our study was to ask whether there are domain-general decision making computations that rely on the similarity between stimuli, *even though* the particularities of how stimuli are mapped into a similarity space may differ between domains.

We agree that there may be some domain differences and inhomogeneity in how stimuli are mapped. Indeed, there is evidence that even 2D spatial environments are not fully homogenous (Bellmund et al., 2020). Previous work using neural imaging has established that there exist representational similarities across domains (e.g., Aronov, Nevers, & Tank, 2017; Constantinescu, O'Reilly, & Behrens, 2016). Yet in these studies homogeneous mapping of stimuli is assumed, not directly shown. As explained above, our

behavioral study was not designed to directly uncover the representational nature of stimuli in different domains, but rather to ask about commonalities and differences in the downstream, behavioral consequences for reward learning and exploration.

Moreover, our task design included a criterion-based pretraining designed to diminish domain differences, and ensured roughly equal exposure to all possible stimuli, resulting in uniform experience of the space. We also designed the input space of the task as a discrete 8×8 grid, where each key press changed the stimuli in a uniform unit of distance in feature space. This fixed travel cost between locations in feature space induces a homogeneous and equivalent relational structure of stimuli across the two domains. This was a defining aspect of our task, which was also reinforced through our training phase. Thus, it is not necessary to test whether moving "one step in 2D frequency/orientation space [is] also one step in the stimulus space of the Gabor stimuli", since this was directly specified by the task structure. We understand that it remains possible that the perceived similarity between stimuli 1 step apart may be different across domains. But we model this potential effect directly using the length-scale of the RBF kernel, which was allowed to differ between domains.

While a pixel-level analysis seems very innovative, we do not think it would be appropriate for our study. Analyzing only the sensory elements of the stimuli (i.e., pixels) would (a) not necessarily reflect the cognitive similarities and (b) assume that the cognitive map ignores transitional structure. We do not feel these assumptions would be justified, based on current theories suggesting that the hippocampal-entorhinal system encodes a combination of both sensory and transitional information (Whittington et al., 2019). Taken together, we believe that the input structure and transition dynamics of the task combined with the learning criterion of the training phase enforce a high standard of consistency across domains.

Nevertheless, we agree that some aspects of our study rationale may not have been clear enough in the initial version of the manuscript. We therefore have added the following statement to the introduction:

> One important implication of these accounts is that reinforcement learning (Sutton & Barto, 1998) in non-spatial domains may rely on a map-like organization of information, supported by the computation of distances or similarities between experiences. These representations of distance facilitate generalization, allowing for predictions about novel stimuli based on their similarity to previous experiences. Here, we ask to what extent does the search for rewards depend on the same distance-dependent generalization across domains, *despite potential differences in how spatial and non-spatial stimuli and their similarities may be processed?* [emphasis indicates added text]

In addition, we believe the Reviewer has raised important outstanding issues. We therefore clarify the limitations of our model and the assumption that we treat each domain equivalently in the general discussion, while acknowledging that representational differences may be a potential explanation for our results:

> Why did we find differences in exploration across domains, even though the tasks were designed to be as equivalent as possible, including requiring commensurate stimuli discriminability during the pre-task training phase? Currently, our model can capture but not fully explain these differences in search behavior, since it treats both domains as equivalent generalization and exploration problems.
>
> One possible explanation is a different representation of spatial and non-spatial information, or different computations acting on those representations. *Recent experimental work has demonstrated that representations of spatial and non-spatial domains may be processed within the same neural systems (Aronov et al., 2017; Constantinescu et al., 2016), suggesting*

representational similarities. But in our study it remains possible that different patterns of exploration could instead result from a different visual presentation of information in the spatial and the non-spatial task. It is, for example, conceivable that exploration in a (spatially or non-spatially) structured environment depends on the transparency of the structure in the stimulus material, or the alignment of the input modality. In our case the spatial structure was embedded in the stimulus itself, whereas the conceptual structure was not. Additionally, the arrow key inputs may have been more intuitive for manipulating the spatial stimuli. While generalization could be observed in both situations, directed exploration might require more explicitly accessible information about structural relationships or be facilitated by more intuitively mappable inputs. [emphasis added to indicate modified text]

And finally, directly discuss inhomogenous mapping as a potential cause of our task order effect:

> *Task order also modulated performance differences between domains, which only appeared when the conceptual task was performed before the spatial task (Fig.2c). Experience with the spatial task version may have facilitated a more homogenous mapping of the conceptual stimuli into a 2D similarity space, which in turn facilitated performance. This asymmetric transfer may support the argument that spatial representations have been "exapted" to other more abstract domains (Hills, 2006; Hills, Todd, & Goldstone, 2008; Todd, Hills, & Robbins, 2012).* For example, experience of different resource distributions in a spatial search task was found to influence behavior in a word generation task, where participants exposed to sparser rewards in space generated sparser semantic clusters of words (Hills, Todd, & Goldstone, 2010). Thus, while both spatial and conceptual knowledge are capable of being organized into a common map-like representation, there may be something special or central about spatial encoding (Nadel, 1991), producing domain differences in terms of the ease of learning such a map and asymmetries in the transfer of knowledge. *Future research should investigate this phenomenon with alternative models that make stronger assumptions about representational differences across domains.* [emphasis added to indicate modified text]

2.3 Other models

> Secondly, as mentioned above, I believe that some attention needs to be paid to other models, like Kohonen maps, e.g., the work in Mok and Love (2019). It might prove useful to give a few sentences on such models' computational properties in order to understand what the paper sets out to investigate: the computational overlap between special and conceptual cognitive processing and what such an overlap might imply. In other words, given the authors are interested in the computational nature of cognitive maps some mention of modelling maps (explicitly) computationally is pertinent. Furthermore, it might help address, or at least contextualise, some of the ideas around the one-directional facilitation effect found and provide a formalisable structure and plan for future work.

Thank you for pointing out the connections offered by the Mok & Love (2019) paper and for the suggestion of adding more connections to other models and theories. In tandem with similar suggestions from Reviewer 3 (see section 4.7), we have added an additional subsection to the general discussion titled "Related work". We reproduce the relevant additions to the text below:

> [...] Recent work building on Kohonen maps has also suggested that the distribution of the experienced stimuli in feature space will have implications for the activation profiles of grid cells and the resulting cognitive map (Mok & Love, 2019).

[...]

Additionally, clustering methods (e.g., Mok & Love, 2019) can also provide local approximations of GP inference by making predictions about novel options based on the mean of a local cluster. For instance, a related reward-learning task on graph structures (Wu, Schulz, & Gershman, 2020) found that a *k*-nearest neighbors model provided a surprisingly effective heuristics for capturing aspects of human judgments and decisions. However, a crucial limitation of any clustering models is it would be incapable of learning and extrapolating upon any directional trends, which is a crucial feature of human function learning (Griffiths, Lucas, Williams, & Kalish, 2009; Lucas, Griffiths, Williams, & Kalish, 2015). Alternatively, clustering could also play a role in approximate GP inference (Liu, Ong, Shen, & Cai, 2020), by breaking up the inference problem into smaller chunks or by considering only a subset of inputs. Future work should explore the question of how human inference scales with the complexity of the data.

2.4 Claims

Thirdly, if all the above is addressed, I would be more comfortable with the claims that this is a "fundamental difference in how people represent or reason about spatial and conceptual domains" but still not completely. Arguably people have vastly more experience with a 2D spatial domain than the domain of Gabor patches. Even the input modality is more easily mappable onto the spatial than conceptual domain since participants used the keyboard arrows in both tasks. I do not believe this is a fatal flaw in the paper, but it is something that has to be touched on: input space and task space are aligned more so in the spatial case than the concept case.

Thank you for pointing out some very thoughtful and theoretically engaging aspects of our paper. We agree that "fundamental" is perhaps too strong of a wording here. We merely wanted to highlight that this reduction in search distance was not due to a lack of effort, but rather coincided with longer search trajectories. We have amended the text to the following:

Whereas participants engaged in typical levels of directed exploration in the spatial domain (replicating previous studies, e.g., Schulz, Wu, Ruggeri, & Meder, 2019; Wu, Schulz, Speekenbrink, Nelson, & Meder, 2018), they displayed reduced levels of directed exploration in the conceptual task, substituting instead an increase in undirected exploration. Again, this is not due to a lack of effort, because participants made substantially longer search trajectories in the conceptual domain (see Fig S4a). Rather, this indicates a *meaningful* difference in how people represent or reason about spatial and conceptual domains in order to decide which are the most promising options to explore. [emphasis added to highlight changed text]

Regarding your comment about people having less experience with Gabor patches, this is a very good point, but perhaps also true for any other feature space. No domain is more central to the human experience than the spatial world around us. Philosophers such as Immanuel Kant have even argued that representations of space must precede experience, while modern cognitive scientists have suggested a one-directional "exaptation" of spatial representations to more abstract, conceptual domains through evolutionary processes (Hills, 2006; Hills et al., 2008; Todd et al., 2012). Thus, it might be an impractical or perhaps quixotic endeavor to seek out conceptual stimuli that are equally familiar as any spatial stimuli. In addition, there are also other issues with using stimuli that participants have prior and thus heterogeneous

experience with, which is why we decided to use abstract stimuli combined with task-relevant training to avoid external confounds.

Nevertheless, differences in prior experience may have played an important role in the patterns of behavior that we observed. One area where we address this point is in the new "Related Work" section of the General Discussion, where we connect differences in prior experience to different tendencies for reuse of previously learned structure. This is one potential explanation for our asymmetric transfer effect:

> A key question underlying the nature of transfer is the remapping of representations (Sanders, Wilson, & Gershman, 2019; Whittington et al., 2019), which can be framed as a hidden state-space inference problem. Different levels of prior experience with the spatial and conceptual stimuli could give rise to different preferences for reuse of task structure as opposed to learning a novel structure. This may be a potential source of the asymmetric transfer we measured in task performance.

To address your comment about the input space being more mappable to the spatial task, you are certainly correct in this intuition. But this may be an inescapable feature of spatial domains. It is not only philosophers and cognitive scientists who have discovered that thinking spatially is intuitive, but also artists, designers, architects, and engineers. The built world around us is abundant with spatial inputs being used to navigate through other domains: we move from lowercase to uppercase to add emphasis to typed text, we click a left arrow on our browser to navigate back to the previous page, and we move up or down through a hierarchy of folders. Thus, while it is certainly intuitive to move through a 2D space with arrow keys, we also believe that the input space for our Gabor stimuli is similarly intuitive. The up and down keys changed the frequency of stripes (much like one can use vertical arrows to change the number of items in an online shopping basket or the value of a cell in a spreadsheet), while the left and right arrows changed the tilt in the aligned direction.

Yet, while similar, we will not dispute that the spatial inputs were still more aligned than the conceptual inputs. In order to address this point, we have added the following text to the General Discussion:

> Recent experimental work has demonstrated that representations of spatial and non-spatial domains may be processed within the same neural systems (Aronov et al., 2017; Constantinescu et al., 2016), suggesting representational similarities. But in our study it remains possible that different patterns of exploration could instead result from a different visual presentation of information in the spatial and the non-spatial task. It is, for example, conceivable that exploration in a (spatially or non-spatially) structured environment depends on the transparency of the structure in the stimulus material, or the alignment of the input modality. In our case the spatial structure was embedded in the stimulus itself, whereas the conceptual structure was not. Additionally, the arrow key inputs may have been more intuitive for manipulating the spatial stimuli. While generalization could be observed in both situations, directed exploration might require more explicitly accessible information about structural relationships or be facilitated by more intuitively mappable inputs.

2.5 Code

> Code: I am having trouble running your code. I suggest the first step is to tidy up your code according to R best practices and especially in terms of dependencies by including a DESCRIPTION file and name these requirements, explaining how to install them in your README file. Also mention the version of R you used to create and run your codebase. See: `http://r-pkgs.had.co.nz/description.html#dependencies` — as well as: `https://github`

`.com/ropensci/Rcleanandhttps://github.com/ironholds/urltools` as examples of good practice to copy from.

We apologize for the difficulty in running the code. The browser viewable HTML notebooks utilized a relatively new Github.io feature. However, it required a modification of the file structure, which broke the loading of some files. These issues have now been fixed. Additionally, while DESCRIPTION files are an important component of an R package (designed for general purpose usage), building a fully fledged package is outside our current scope. The github repository containing data, code, and interactive notebooks is designed to provide complete and step-by-step instructions for reproducing all analyses conducted in this paper. Indeed, the detail included in the interactive notebook goes well beyond any common standard in open science. Nevertheless, we have also improved the level of documentation and have added the R version number to the README of the code repository.

2.6 Typo

Minor: Figure 3 panel d has a typo, should be "Bonus".

Thank you for pointing this out. The typo has been corrected in the updated version.

3 Reviewer 2

3.1 General remarks

> I enjoyed reading this paper. I think trying to understand computational differences in how individuals reason and generalize in spatial and non-spatial maps explore is an important problem in cognitive science. The study presented in the paper finds some intriguing similarities and differences between generalization and exploration in these domains that I think will be inspiring for future research. In general, the analysis are very well presented and I appreciate the thorough investigation of the behavioral data in a model-free manner in addition to the sophisticated model-fitting. I only have a few critiques.

We thank Reviewer 2 for the positive and valuable feedback.

3.2 Clarification on the training task

> As a side point here, I'm not sure I fully understood the training task and what exactly data from it is meant to show. Was the target on the screen while the subjects navigated to it, or were subjects required to hold the target in memory? Additionally, in order to receive a correct response, were subjects required to take the shortest path to the target, or merely to arrive at the target eventually - perhaps this would bear on whether subjects intuited a map-like distance in the conceptual space that is similar to the map distance in spatial space?

We thank the reviewer for highlighting a part of the manuscript that needed clarification. The training task was designed to familiarize participants with the stimuli and inputs of the experiment. Specifically, we wanted to ensure that participants were able to reach the same level of proficiency with both spatial and conceptual stimuli, in terms of being able to navigate towards and select a target stimuli. As depicted in Fig. 1c (reproduced below as Fig. R2), the target was shown on the screen below the current selection. Participants were thus asked to navigate their selection to match the target and then press spacebar. They did not have to hold the target in memory. We distinguished only between correct and incorrect responses, and not the length of the trajectory.

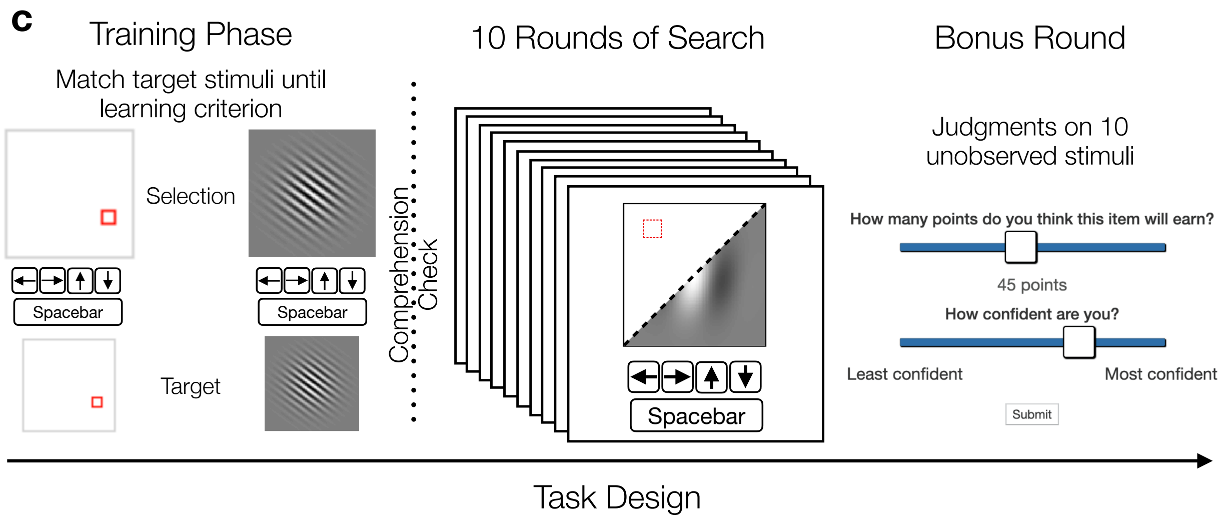

Figure R2: Reproduced from Figure 1c in the main text.

To improve how the training task is communicated, we have amended the description in the introduction to the following:

> To ensure commensurate stimuli discriminability between domains, participants completed a training phase where they were required to reach the same level of proficiency in correctly matching a series of target stimuli (see Methods; Fig. 1c).

In addition, we have also changed the description of the training task in the methods section, to emphasize that the target was shown alongside the participant's current selection:

> Participants were shown a series of randomly selected targets and were instructed to use the arrow keys to modify a single selected stimuli (i.e., adjusting the stripe frequency and angle of a Gabor patch or moving the location of a spatial selector, Fig.1c) *in order to match a target stimuli displayed below. The target stayed visible during the trial and did not have to be held in memory.* [Emphasis indicates changed text]

3.3 Distance dependent generalization

> Comparisons in generalization and exploration parameters between the two tasks rely on distances between the two stimuli meaning the same thing between the two tasks. There's no reason though that this should inherently be the case. The authors use evidence from the training task to argue that there are not perceptual discriminability differences between the stimuli for the two tasks. I'm not sure I understood though how this would address the question of whether distances between stimuli are comparable.
>
> Related to the question about how we can know whether distances between tasks are equivalent, I'm not sure it's fair to assume that distances across the two dimensions of the conceptual stimuli mean the same thing (as i think is assumed by the RBF kernel in the GP model.) I think the authors should either address this with further analysis, or discuss whether this assumption being untrue would change interpretation of parameters from the model.
>
> If it is not possible to address these concerns with further analysis, I think the paper is still valuable and interesting, but I think the authors should address, in the discussion, whether this concern poses limitations in the interpretation of parameter similarities and differences.

This is a very excellent question and addresses an important theoretical aspect of our model. The reviewer is certainly correct that we have no reason to believe *a priori* that the same unit of distance is used across domains. However, the length-scale parameter λ is specifically designed to control for different scales of distance in different contexts. We provide the intuition in the text that λ controls the extent of generalization, where larger values indicate broader generalization. Yet another interpretation is that λ controls the scale at which we interpret stimuli distance, where larger values indicate a larger shrinking of distance. Thus, our within-subject comparison of the length-scale (λ) estimates provided empirical evidence that were no systematic differences between spatial or conceptual tasks. This suggests that the extent of generalization (relative to whichever unit of distance) was the same across domains.

It is also important to point out a crucial layer of context to this lack of difference in length-scale: in both domains, the search space was discretized into the same number of units, movement had the same action costs (one key press per unit of movement), and we provided participants with the same goals across tasks (both in the training and bandit task). These may have all been important factors that contributed to our result. In the real world, where there are no such experimental controls and uniform discretization, there may be natural differences in the perceived scaling of distance, which we could potentially capture as differences in λ estimates.

3.4 Scope of behavioral analyses explained by the GP

I appreciated the in depth model-free analysis (Behavioral Results section) prior to the modeling analysis. However it seems that a number of the features of the data that are presented in the model-free are not addressed again in the modeling section. This leads to the impression that perhaps there are aspects of behavior that the GP model is not picking up.

In particular, I was wondering whether differences in model parameters (between environments and also between tasks) can account for the following features in the data:

- That participants get more rewards in smooth compared to rough domains

- The one-directional transfer effect that subjects conceptual performance benefits from first performing the spatial task.

We thank the Reviewer for the positive feedback. In regards to the scope of our model, the GP is in fact able to capture the relative differences between environments in the learning curve simulations. We provide an aggregation of the simulated learning curves from Fig. 3c below in the form of a bar graph (Fig. R3), in order to better illustrate the relative differences in performance across environments. Although we did not find direct evidence of environmental differences when comparing λ estimates, our simulated data generated from these participant parameter estimates revealed qualitatively different pattern of performance, which replicated the pattern from our participants.

Whereas the GP and Human subjects both perform better in smooth environments, the BMT performed slightly better in the rough environment, echoing a similar difference in the random baseline[1]. Note that error bars are not added, because the model data is aggregated over 10k simulations, making any perceivable differences statistically significant.

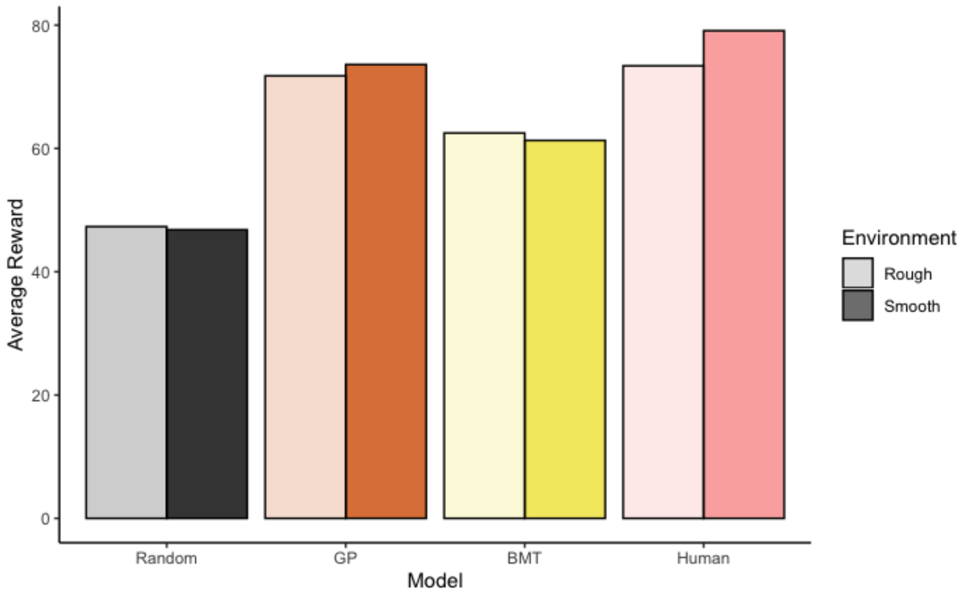

Figure R3: Model simulations across environments. With the exception of the human data from the experiment, the height of each bar indicates the average reward over 10k simulations generated from participant parameter estimates.

[1]The differences in random performance is due to sampling bias in the generation of the set of 40 smooth and 40 rough environments used in both the experiment and simulations. The generative model was defined with the same expected reward across environments, however it is reasonable to have some variation in an empirical sample of 40 environments of each class.

Regarding the one-directional transfer effect, we have amended the discussion to clarify that this was not within the scope of our model:

> Another interesting finding is the one directional transfer from the spatial to the conceptual domain, *which was not accounted for by our model.... Future research should investigate this phenomenon with alternative models that make stronger assumptions about representational differences across domains.* [Emphasis indicates modified text]

3.5 Differences in GP learning curves across environments

> Relatedly, in comparing human and model learning curves (figure 3c) it appears that humans outperform the model in smooth, but not in rough environments. Why does the model fail to capture learning curves as well in smooth environments?
>
> I think it is fine if the model cannot account for all these differences. But it would be useful for the reader for the paper to clearly state what aspects of the model-free analysis the GP models can and cannot account for.

The reviewer is correct that the simulated learning curves are closer to human performance in smooth environments compared to rough environments. This is particularly the case in the early trials of the smooth environments. However, as shown in Figure R3 (above), the GP nevertheless displays the same qualitative difference between environments as humans, with better performance in the smooth environment. This was not the case for the BMT and random simulations. We have also replicated this analysis comparing the simulated performance across domains (Fig. R4). Again, the GP reproduces the same pattern of behavior, with better performance in the spatial task, whereas the BMT and random models display equivalent performance.

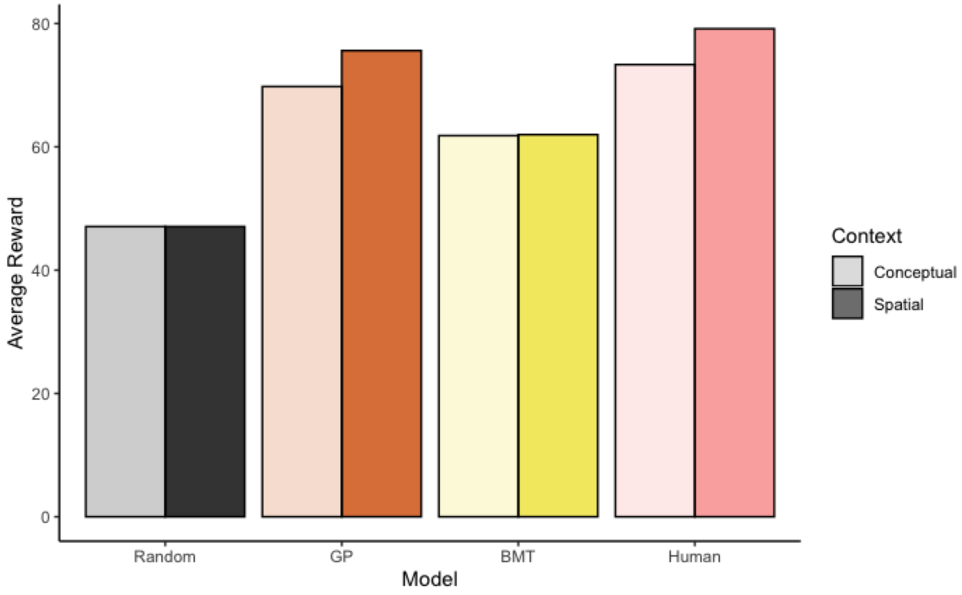

Figure R4: Model simulations across domains. The height of each bar indicates the average reward over 10k simulations (with the exception of the human data).

Since simulated performance is dependent on participant parameter estimates used to specify the models, these differences in performance are largely driven by the strong differences in directed exploration β

and random exploration τ parameters. Taken together, we believe that our current description of the GP as producing "realistic learning curves" is justified by the results. In order to make this comparison more salient, we have added a new plot to Figure 3 (inset shown below as Fig. R5; Fig. 3d in the main text) and have added the following text to the results:

> In addition, the GP captured the same qualitative difference between domains and environments as human (Fig. 3d), with better performance in conceptual vs. spatial, and smooth vs. rough. These patterns were not present in the BMT or random simulations.

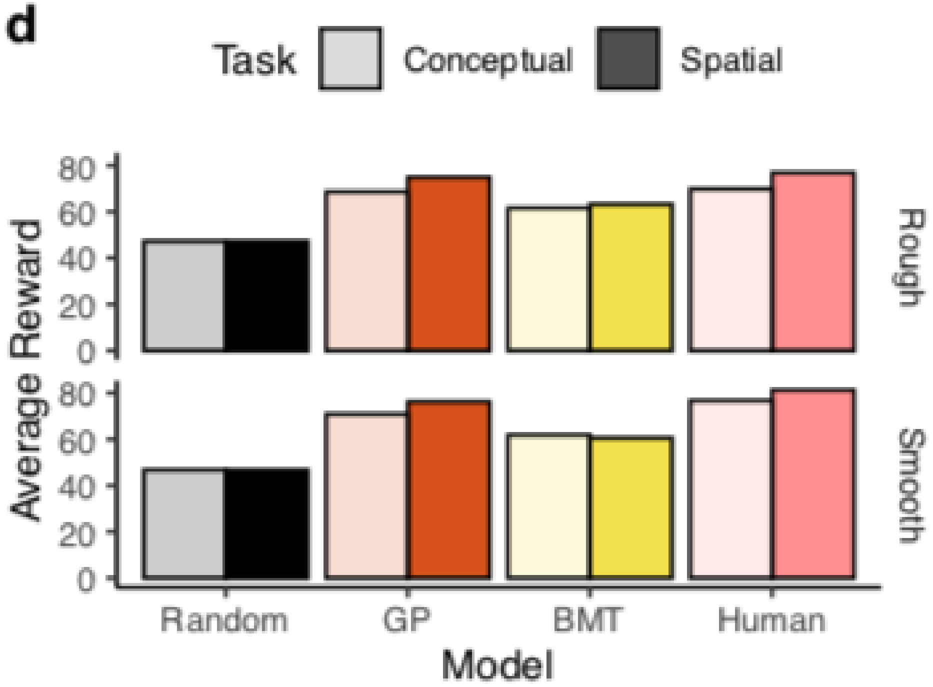

Figure R5: New panel d added to Figure 3 in the main text. Simulation results aggregated over trials, where the height of the bar indicates average reward.

3.6 Differences in exploration parameter

> Lastly, for comparison of exploration parameters, I think the difference in directed exploration between environments is a really interesting difference between tasks. However, I question whether it is fair to interpret differences in the random exploration parameter as a strategy difference. This is because, I presume, that errors in model prediction of behavior get soaked up into that parameter. If this is correct, couldn't the paper equivalently just state that the model fits worse in the conceptual task than the spatial task?

The reviewer makes an important point by raising this issue with the random exploration parameter potentially soaking up errors in model prediction. We would first like to be more precise about what we mean by random exploration, and we then show that the data supports our argument of there being a difference exploration rather than merely a difference in model fit.

First of all, we can look at how the temperature parameter τ changes across tasks while controlling for differences in model fit using a Bayesian regression. Specifically, we predicted $\log(\tau)$ using

task and predictive accuracy of the model (R^2) as predictors, along with their interaction term, while using participant id as a random intercept. We visualize below the conditional effects of task on τ, which shows that this difference still emerges accounting for model fit. This analysis has been added to the following section of the online supplement: `https://charleywu.github.io/cognitivemaps/modelingResultsNotebook.html#relationships-between-parameters-and-predictive-accuracy`.

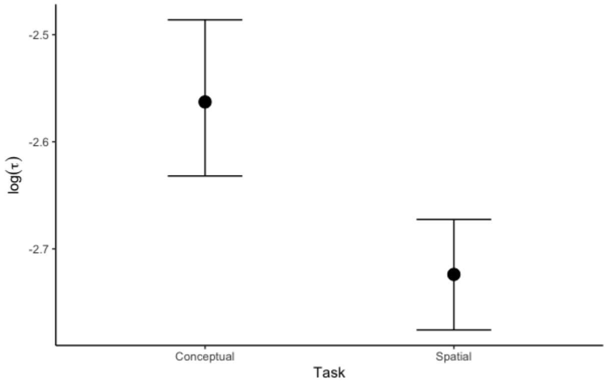

Figure R6: Conditional effect of task on the estimated temperature parameter τ, where error bars indicate the 95% credible interval.

Additionally, we have also replicated the same change in directed exploration parameters for the BMT (Fig. S9) and for the Shepard kernel models (Fig. S10). Thus, this consistent change in random exploration across models, is indicative of a broader pattern of results, instead of model-specific prediction error. We have aggregated plots from Fig. 3e, Fig. S9 and Fig. S10 below for ease of comparison.

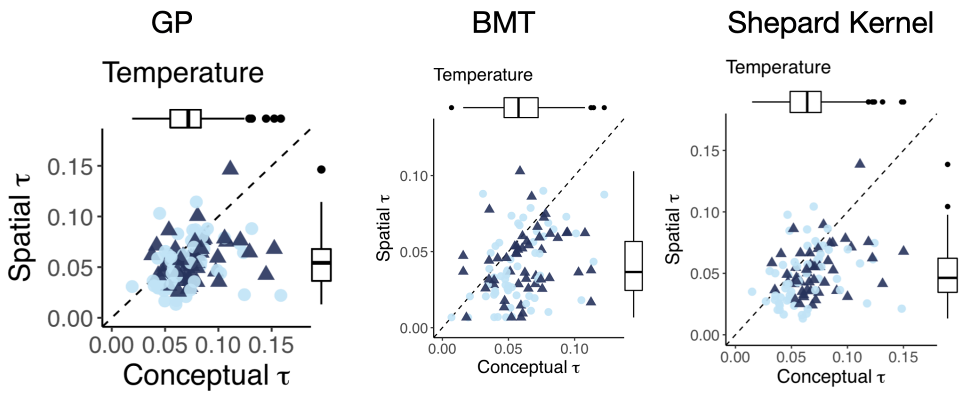

Figure R7: Comparison of temperature parameters across models. Aggregated from Fig. 3e, Fig. S9 and Fig. S10.

Lastly, because the reviewer makes an excellent point that τ can in principle soak up errors in model prediction, we added some clarification to our interpretation of the results. It is true that random exploration is not necessarily an overt strategy, but may also reflect various forms of decision noise. For instance, in a recent paper, Findling, Skvortsova, Dromnelle, Palminteri, and Wyart (2019) show that random exploration can be caused by computational noise due to limited precision during the learning

of action values, which draws an important connection between random exploration and computational demands. We have added the following text to our discussion to address this point:

> Nevertheless, differences in random exploration could also arise from limited computational precision during the learning of action values (Findling et al., 2019). Thus, the change in random exploration we observed may be due to different computational demands across domains.

4 Reviewer 3

4.1 General remarks

> Wu and colleagues tested human participants on a spatial and conceptual task to assess whether similar cognitive mechanisms are used across these domains. They applied computational modelling and found shared and different processes, suggesting some processes are shared whereas other processes might be distinct.
>
> This is an exceptionally well conducted study with a clear rationale, strong analyses and modelling work. I believe this will be a great paper for PLOS computational biology, after minor revisions. Mainly, I have some questions to clarify parts of the paper, and on how the works compares to some of the current literature. I also include some few suggestions that I hope will help the paper, if space allows.

We thank Reviewer 3 for the positive and thoughtful feedback.

4.2 Differences between environments

> Smooth vs rough designs: Is the reason why people get more rewards on smooth conditions because there is more rewards overall across the map, or is it actually that they do better in smooth environments because they learnt it?
>
> I ask because it looks like smooth environments have more rewards (looking at the maps in S2 - more yellow cells). But it also sounds like it is normalized so overall same expected reward - so would that mean each yellow cell means lower rewards in the smooth versus rough? But if that's the case then it's much harder to get higher reward in the smooth conditions (since highest reward per choice is lower?)

Both environments have the same expected rewards across options. This is described in the first paragraph of the results section, which we have modified to clarify this point:

> The strength of reward correlations were manipulated between subjects, with one half assigned to *smooth* environments (with higher reward correlations) and the other assigned to *rough* environments (with lower reward correlations). Importantly, both classes of environments had the same expectation of rewards across options. [Emphasis in original]

Thus, we can confidently make the claim that participants performed better in smooth environments by virtue of generalization, and not due to differences in the overall reward distribution. To clarify this point, we have added the following text to the caption in Fig. S2:

> All environments have the same minimum and maximum reward values, and the two classes of environments share the same expectation of reward across options.

4.3 Attention to feature dimensions and transfer

> Do participants attend to one feature dimension more than another (e.g. Nosofsky, 1986 - you could check if they weighted one dimension more than another / more sensitive to one dimension)? Probably not for spatial, but maybe in the gabors? Does that affect anything / maybe harder to generalize to space if so?

Related to the above point - transfer - is there an analysis to show why there is transfer for space –> gabors but not vice versa? E.g. general: people who learn better on space –> transfer more. If so - maybe people who learn better on gabors also transfer more to space (even though main effect not there). Possibly, those who show more 'equal' attention to both dimensions on gabors show better transfer?

We thank the reviewer for raising an excellent point and for suggesting an analysis we had not considered. We address this comment through several behavioral and model-based analyses.

Behavioral analyses of attentional biases

First, we analyzed participant search trajectories by decomposing them into the vertical/stripe frequency dimension vs. horizontal/tilt dimension. Intuitively, this is a relative measure of the number of times participants pressed the up/down arrows vs. the left/right arrows to manipulate the stimuli before making a selection. This provides an approximate measure of the amount of attention participants paid to a specific dimension, by capturing the quantity of interactions with each dimension. Figure R8 below (added as Fig. S4d in the manuscript) shows the proportion of participant inputs corresponding to each dimension, where we see an higher proportion of inputs given to the vertical/stripe frequency dimension in both tasks, relative to the horizontal/tilt dimension.

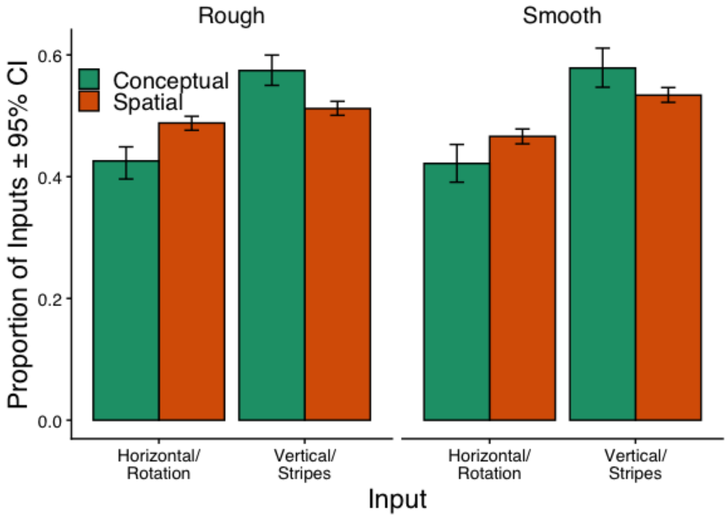

Figure R8: Search trajectories decomposed into the proportion of inputs given along the vertical/stripe frequency dimension vs. horizontal/tilt dimension. Bars indicate group means and error bars show the 95% CI. Added to the manuscript as Fig. S4d.

We formally define differences in attention $\Delta_{\text{dim}} = P(\text{vertical/stripe frequency}) - P(\text{horizontal/tilt})$, where positive values indicate a stronger bias towards the vertical/stripe frequency dimension. We then ran a two-way mixed ANOVA comparing the influence of task and task order on Δ_{dim} (Fig. R9; Fig. S4e in the manuscript)[2]. We found a strong interaction between task order and task ($F(1,127) = 8.1$, $p = .005$, $\eta^2 = .02$, $BF > 100$). While participants were more biased towards the vertical/stripe frequency dimension in the conceptual task when the conceptual task was performed first ($t(66) = -6.0$, $p < .001$,

[2]We found no direct or interaction effects of environment on Δ_{dim}, and thus removed it as a predictor.

$d = 0.7$, $BF > 100$), these differences disappeared when the spatial task was performed first ($t(61) = -1.6$, $p = .118$, $d = 0.2$, $BF = .45$).

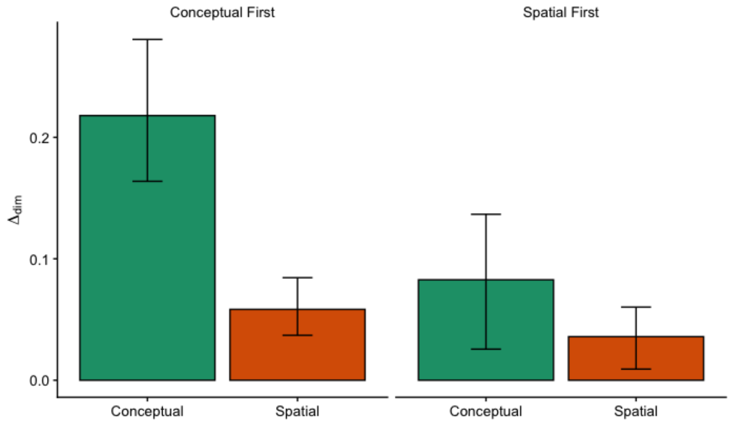

Figure R9: Attentional bias Δ_{dim}. Positive values of Δ_{dim} indicate a bias towards the vertical/stripe frequency dimension, while negative values indicate a bias towards the horizontal/tilt dimension. The height of the bar indicates the group mean, while the error bar shows the 95% CI. Added to the manuscript as Figure S4e.

Next, we looked at whether attentional differences were also predictive of performance. The results are presented in Figure R10, where each pair of dots is a single participant, and the connecting line shows the change in score and change in attentional bias Δ_{dim} across tasks. We find a negative relationship between score and attention for the conceptual task only in the conceptual first order ($r_\tau = -.31$, $p < .001$, $BF > 100$), but not in the spatial first order ($r_\tau = -.07$, $p = .392$, $BF = .24$). There were no relationships between score and attention in the spatial task in either order (spatial first: $r_\tau = .03$, $p = .738$, $BF = .17$; conceptual first: $r_\tau = -.03$, $p = .750$, $BF = .17$). Thus, strong attentional biases were associated with lower score, but only in the conceptual first task order and for the conceptual task.

We now look at differences in attentional biases between tasks (Fig R11). We define $\Delta_{task} = \Delta_{dim}^{Spatial} - \Delta_{dim}^{Conceptual}$. This difference of differences is a bit more difficult to interpret, but recall that Δ_{dim} tended to be positive, since participants attended more towards the vertical/stripe frequency dimension in both tasks. Thus, Δ_{task} is *positive* if participants were more biased towards the vertical/stripe frequency dimension in the spatial task. Vice versa, Δ_{task} is *negative* if participants were more biased towards the vertical/stripe frequency dimension in the conceptual task.

Figure R11 shows the relationship between Δ_{task} and change in the difference in score between tasks. Looking first at participants in the Conceptual First condition, we find an anecdotal relationship between Δ_{task} and difference in score ($r_\tau = -.20$, $p = .019$, $BF = 2.4$). The directionality of this effect is that participants with a stronger bias towards the vertical/stripe frequency dimension in the conceptual task (i.e., negative Δ_{task}) tended to have a lower score in the conceptual task relative to the spatial task – albeit with only weak evidence. We find no relationship between Δ_{task} and difference in score for the Spatial First condition ($r_\tau = -.04$, $p = .666$, $BF = .18$). An outstanding question is whether this shift in attention is responsible for the transfer effect, or is merely an artifact of participants being more adept at navigating the full search space in the conceptual domain after prior experience with the spatial tasks. While these analyses provide further clarification about the role of attention, the exact relationship between attention (as measured by input frequency) and generalization is outside the scope of our current paper. Future work using alternative manipulations and measurements (e.g., eye-tracking) would be better suited for

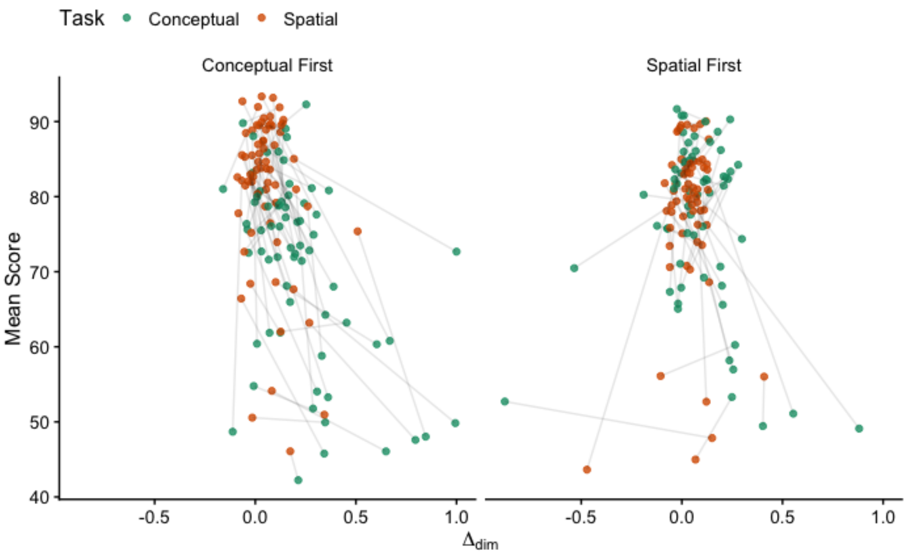

Figure R10: Relationship between within-task attentional bias Δ_{dim} (x-axis) and mean score. Positive values of Δ_{dim} indicate a bias towards the vertical/stripe frequency dimension, while negative values indicate a bias towards the horizontal/tilt dimension. Each participant is described by a pair of connected dots, where colors indicate the task. Added to the manuscript as Figure S4f.

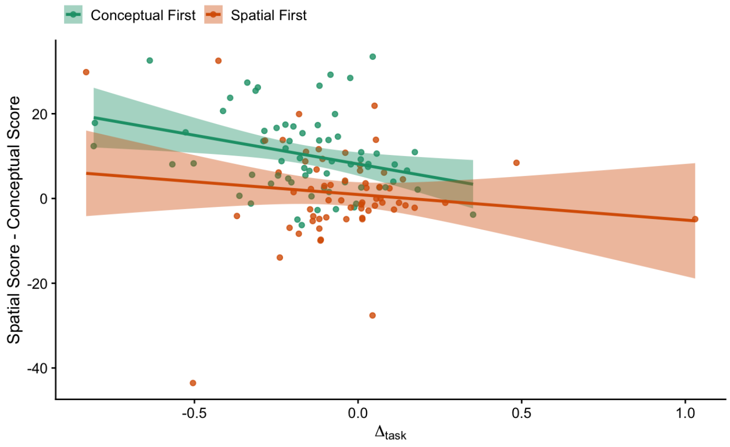

Figure R11: Change in score as a function of change in attentional biases. Δ_{task} is positive when participants were more biased towards the vertical/stripe frequency dimension in the spatial task, and is negative when participants were more biased towards the vertical/stripe frequency dimension in the conceptual task. Each point can also be interpreted as the slope from Fig. R10, and thus has not bee included in the manuscript.

answering this question.

Thus, as an interim conclusion to these behavioral analyses, we found i) differences in attentional bias across domains, ii) this bias is influenced by task order, and iii) high bias predicted lower scores in the Conceptual First condition. We also found anecdotal evidence showing increased bias towards the vertical/stripe frequency dimension in the conceptual task was associated with a larger gap in score. We have added three new figures (Figs. S4d-f), with statistical tests described in the figure captions in

order to address the role of attentional biases in our experiment. We have added new text to the general discussion to highight these analyses (discussed below at the conclusion of this section) and have included the code used to reproduce these analyses in the online supplement (`https://charleywu.github.io/cognitivemaps/behavioralResultsNotebook.html`).

Model-based analysis of dimension integration

We now turn to a model-based analysis for another perspective on this current question, by examining whether there exist domain differences in how people integrate the separate dimensions (Austerweil, Sanborn, & Griffiths, 2019). This analysis uses the Shepard kernel (Jäkel, Schölkopf, & Wichmann, 2008) as an extension of the RBF kernel we report in the main text. Rather than defining similarity using squared Euclidean distance, the Shepard kernel uses Minkowski distance:

$$k_{Shepard}(\mathbf{x}, \mathbf{x}') = \frac{\left(\sum_i |x_i - x_i'|^{\rho}\right)^{1/\rho}}{2\lambda^2}, \quad (4)$$

where the order parameter $\rho \leq 2$ is an additional free parameter that we estimate. The RBF kernel is thus a special case of the Shepard kernel, where $\rho = 2$. When the Minkowski distance parameter $\rho < 2$, the implication is that the separate dimensions are less than fully integrated with each other, suggesting people are generalizing more independently along the two dimensions. Estimates for the Shepard kernel are reported in Fig. S10 (reproduced below), where we do not find robust differences in ρ across domains, nor do we find correlated estimates between tasks. Thus, this model analysis provides support that there are no systematic differences in how people integrate the two feature dimensions between spatial and conceptual tasks. Moreover, we recover the same main findings from the Shepard kernel, with respect to the change in directed and random exploration. We report the more intuitive RBF kernel in the main text, while keeping the Shepard kernel in the supplementary materials and online supplement.

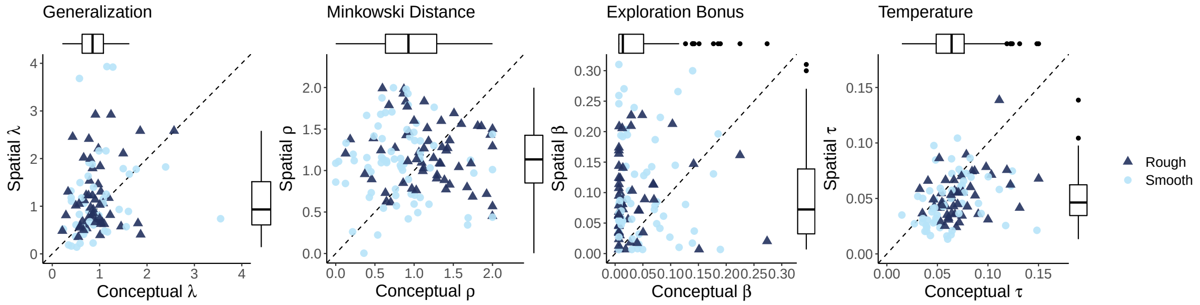

Figure R12: Shepard kernel parameters. Reproduced from Fig. S10

In conclusion, our results indicate some bias in how people attend to the separate feature dimensions and less than perfect integration across features (i.e., $\rho < 2$). To address this comment, our manuscript provides both behavioral (Fig. S4d-f) and model-based analyses (Fig. S10) to present these relationships. We have added text in the discussion to highlight these analyses (below). However, the interpretation of our main findings remains unchanged.

> Our model also does not account for attentional mechanisms (Radulescu, Niv, & Ballard, 2019) or working memory constraints (Collins & Frank, 2018; Ohl & Rolfs, 2018), which may play a crucial role in influencing how people integrate information differently across domains. Indeed, the "stretchy birds" paradigm used by (Constantinescu et al., 2016) as

evidence for a common neural representation of spatial and conceptual knowledge required several hours of training before being measured in the scanner. *In our experiment we found differences in attention given to the separate feature dimensions, which may have contributed to our transfer effect (Fig. S4d-f). We also implemented a variant of our GP model using a Shepard kernel (Jäkel et al., 2008) to model domain differences in feature integration (Austerweil et al., 2019), which failed to reveal any coherent patterns (Fig. S10).* Alternatively, differences in random exploration could also arise from limited computational precision during the learning of action values (Findling et al., 2019). Thus, the change in random exploration we observed may be due to different computational demands across domains. Similar shifts increases to random exploration have also been observed under direct cognitive load manipulations, such as by adding working memory load (Cogliati Dezza, Cleeremans, & Alexander, 2019) or by limiting the available decision time (Wu, Schulz, Gerbaulet, Pleskac, & Speekenbrink, 2019). [emphasis added to indicate relevant changes in the text]

4.4 Model comparison

The results of 3A are convincing but shows that the BMT model does well as well; whereas 3B looks like it's doing very poorly; why is this? I realize A is predicting novel choices, and B is model fit. Is BMT actually doing a very bad job of fitting, but still getting pretty good predictive accuracy?

Figure 3a and 3b are different representations of the same underlying model fits, based on the total out-of-sample log loss. Fig. 3a reports each individual estimate in terms of predictive accuracy (Eq. 14), providing an intuitive pseudo-R^2 representation of the objective model fits.

While R^2 values make the objective performance of the models more intuitive, it can be difficult to compare the relative differences between models for any given participant. The data distributions in Fig. 3a may appear quite similar, yet even a paired t-test reveals strong and reliable differences. However, these differences are masked by the heterogeneity across participants.

For this reason, we use a Bayesian model selection framework designed for group studies (Rigoux, Stephan, Friston, & Daunizeau, 2014; Stephan, Penny, Daunizeau, Moran, & Friston, 2009). This is reported as the protected exceedence probability (pxp), which intuitively, can be described as a random-effect analysis. Models are treated as random effects and are allowed to differ between subjects, where we assume that there is a fixed but unknown distribution of models in the population. The bars in Fig. 3b thus describe the posterior probability (corrected for chance) that a given model is more frequent in the population than all other models in consideration.

In order to clarify that both Fig. 3a and 3b are different interpretations of the same models fits, we have modified the text where we introduce the pxp results:

Comparing this same out-of-sample prediction accuracy using a Bayesian model selection framework (Rigoux et al., 2014; Stephan et al., 2009) confirmed that the GP had the highest posterior probability (corrected for chance) of being the best model in both tasks (protected exceedance probability; conceptual: $pxp(\text{GP}, \text{conceptual}) = .997$; spatial: $pxp(\text{GP}, \text{spatial}) = 1.000$; Fig 3b). [Emphasis added to mark changes]

4.5 Mismatch in reward structure

Do the rewards have to be spatially correlated for the GP model to work well / generalize? Comparing the BMT (point estimate) versus the GP model - it makes sense that the function

learning approach will do better than a point estimate approach when locations in the space are correlated. Would your approach do as well if they were less/not (spatially) correlated? i.e. would the GP model still work with learning structures that were not, e.g. distributed smoothly?

My question is - if there is structure, but not spatially smooth at all (rough is still quite smooth), would the GP still learn it? Eg. Across blocks, you change the rewarded locations, but the relations between the reward locations are kept the same. Or would a point-estimate model do as well?

It is not the case that rewards have to be spatially correlated in the strict sense. From a purely technical perspective, a GP is capable of learning any stationary function (Rasmussen & Williams, 2006). From a psychological perspective, previous work has found evidence that the GP can also predict behavior using unsmooth environments. For instance, Experiment 3 in Wu et al. (2018) used a set of natural environments based on agricultural data. The task in that paper was similar, but the bandit was defined as a 11×11 grid and participants clicked on individual tiles using the mouse instead of the navigational inputs (arrow keys and spacebar) used here. There, the rewards were defined by the normalized yield of various crops (e.g., lemons, pumpkins, wheat, etc...), where the rows and columns of the grid corresponded to the rows and columns of an experimental farm. These environments had some level of spatial correlation, but were highly heterogeneous both within and across environments. Nevertheless, in those unsmooth environments and compared amongst a set of 27 different models, GP-UCB was still able to capture and simulate human-like performance.

We would also expect the GP to provide accurate predictions of human behavior in the extreme case of independent reward distributions. As $\lambda \to 0$, the GP would learn the rewards of each option independently and mimic the behavior of the BMT model. This special case applies to the typical multi-armed bandit context, where rewards for each arm are normatively assumed to be independent and where the BMT has been widely used as a model of human behavior (Acuna & Schrater, 2008; Gershman, 2018; Speekenbrink & Konstantinidis, 2015; Wu et al., 2019). Thus, the GP provides a good description of human performance amongst a wide range of environments.

4.6 Other models and theoretical ideas

The authors only compare with one model - BMT, a Bayesian model that does point estimates on rewards. How about other models? The claim that GP is a good model because it captures the generalization bit is fine - but maybe less emphasis on the model as the only model? E.g. can other models solve it? Maybe there are good reasons this model is better theoretically anyway - could discuss this and why it's better/ different to other models at least

Theoretically, would a gaussian mixture model or clustering model not also work for structures where the rewards are spatially correlated like this? You'd get generalization to new parts of the space if they learn the centres of the reward regions - though I'm not sure if it would learn as quickly as is needed (compared to GPs)

To be clear, I am not asking the authors to run all the models, but state what they show (they can show good generalization, but not that this is the only model can do that). It could make sense to discuss other models and maybe why they won't work if that is the case.

[....] How is it related to other ideas about the neural underpinnings, e.g. grid cells? For example, the successor representation (e.g. Stachenfeld et al., 2017, Mommenejad & Howard, 2018), clustering (Mok & Love, 2019), Gaussian/Bayesian mixture models and more (e.g.

> Sanders, Wilson, Gershman 2019, bioRxiv), spatial-conceptual (Bellmund et al., 2019- cited but relevant comparison). Would these models do well at your task, or is GP the only one that could capture the data? Are there any predictions or interpretations of the GP for neural data?

We thank the reviewer for this thoughtful feedback. Clustering models may be capable of approximating generalization at a very local level, yet all predictions about observed rewards would be regressed to the mean of the cluster. Thus, any clustering approach would fail to learn any directional trends. For instance, having observed a pattern of gradually increasing reward towards a corner of the search space, a clustering model would predict that the next option in that direction would be equal to the mean of the cluster, rather than continuing the trend.

In contrast, a long line of research on humans learn functions (Carroll, 1963; DeLosh, Busemeyer, & McDaniel, 1997; Kwantes & Neal, 2006) has found that extrapolation based on inferred patterns is a central phenomena. It is in this domain of explicit function learning that the GP emerged as a candidate psychological model of human learning (Griffiths et al., 2009; Lucas et al., 2015), capable of replicating most of the observed empirical phenomena of human function learning.

However, we agree that we should be careful to qualify our statements about the GP being the best model, and to discuss other models that can also provide mechanisms for generalization. To address this concern, which was also shared by Reviewer 1, we have added an additional section to the general discussion to describe related models and theories:

> ## Related work
>
> Our findings also contribute to a number of other cognitive models and theories. According to the successor representation (SR; Dayan, 1993) framework, hippocampal cognitive maps reflect predictions of expected future state occupancy (Bellmund et al., 2020; Russek, Momennejad, Botvinick, Gershman, & Daw, 2017; Stachenfeld, Botvinick, & Gershman, 2017). This provides a similarity metric based on transition dynamics, where an analytic method for computing the SR in closed form is to assume random transitions through the state space. This assumption of a random policy produces a nearly identical similarity metric as the RBF kernel (Wu et al., 2020), with exact equivalencies in certain cases (Machado et al., 2018).
>
> However, the SR can also be learned online using the Temporal-Difference learning algorithm, leading to asymmetric representations of distance that are skewed by the distance of travel (Mehta, Quirk, & Wilson, 2000; Stachenfeld et al., 2017). Recent work building on Kohonen maps has also suggested that the distribution of the experienced stimuli in feature space will have implications for the activation profiles of grid cells and the resulting cognitive map (Mok & Love, 2019).
>
> In our current study, we have focused on the simplifying case of a cognitive map learned through a random policy. This context was induced by having stimuli uniformly distributed over the search space and using a training phase involving extensive and random trajectories over the search space (i.e., matching random targets from random starting points). While this assumption is not always met in real life domains, it provides a useful starting point and allows us to reciprocally compare behavior in spatial and conceptual domains.
>
> Previous work has also investigated transfer across domains (Mark, Moran, Parr, Kennerley, & Behrens, 2019), where inferences about the transition structure in one task can be generalized to other tasks. Whereas we used identical transition structures in both tasks, we nevertheless found asymmetric transfer between domains. A key question underlying the

nature of transfer is the remapping of representations (Sanders et al., 2019; Whittington et al., 2019), which can be framed as a hidden state-space inference problem. Different levels of prior experience with the spatial and conceptual stimuli could give rise to different preferences for reuse of task structure as opposed to learning a novel structure. This may be a potential source of the asymmetric transfer we measured in task performance.

Additionally, clustering methods (e.g., Mok & Love, 2019) can also provide local approximations of GP inference by making predictions about novel options based on the mean of a local cluster. For instance, a related reward-learning task on graph structures (Wu et al., 2020) found that a *k*-nearest neighbors model provided a surprisingly effective heuristics for capturing aspects of human judgments and decisions. However, a crucial limitation of any clustering models is it would be incapable of learning and extrapolating upon any directional trends, which is a crucial feature of human function learning (Griffiths et al., 2009; Lucas et al., 2015). Alternatively, clustering could also play a role in approximate GP inference (Liu et al., 2020), by breaking up the inference problem into smaller chunks or by considering only a subset of inputs. Future work should explore the question of how human inference scales with the complexity of the data.

Lastly, the question of "how the cognitive map is learned" is distinct from the question of "how the cognitive map is used". Here, we have focused on the latter, and used the RBF kernel to provide a map based on the assumption of random transitions, similar to a random-policy implementation of the SR. While both the SR and GP provide a theory of how people utilize a cognitive map for performing predictive inferences, only the GP provides a theory about representations of uncertainty via Bayesian predictions of reward. These representations of uncertainty are a key feature that sets the GP apart from the SR. Psychologically, GP uncertainty estimates systematically capture participant confidence judgments and provides the basis for uncertainty-directed exploration. This distinction may also be central to the different patterns of search we observed in spatial and non-spatial domains, where a reduction in uncertainty-directed exploration may also reflect computational differences in the structure of inference. However, the nature of these representations remains an open question for future neuroimaging research.

4.7 Suggestion for abstract

Key findings include both similarities and differences between cognitive mechanisms for the two tasks - the differences are described well but the similarities are a bit vague: "Using a Bayesian learning model, we find evidence for the same computational mechanisms of generalization across domains." If there is space, I suggest the authors could add a sentence or state what they find - that there are no/little differences between the parameters from these model across tasks, and they are correlated across participants (or qualify which parameters were not different/correlated).

We thank the Reviewer for this clear and well articulated suggestion. We have added the following text in the abstract to emphasize the lack of difference in model estimates of generalization:

The same Gaussian Process model best captured human search decisions and judgments in both domains, and could simulate realistic learning curves, *where we found equivalent levels of generalization in spatial and conceptual tasks.* [emphasis added to indicate changed text]

We also felt that this point was not made clearly enough in the general discussion, and made similar changes there as well

> *In both domains, our parameter estimates indicated equivalent levels of generalization. Using these parameters, our model was able to simulate human-like learning curves and make accurate out-of-task predictions about participant reward estimations and confidence ratings in a final bonus round. This model-based evidence for similar distance-based decision making in both domains was also in line with our behavioral results.* Performance was correlated across domains and benefited from higher outcome correlations between similar bandit options (i.e., smooth vs. rough). Subsequent choices tended to be more local than expected by chance, and similar options where more likely to be chosen after a high reward than a low reward outcome. [emphasis added to indicate changed text]

4.8 Suggestions for introduction

> - An explanation of what 'generalization' in cited work and in the current work is assumed. Something simple would already help: e.g. when people learn or gain an understanding about an environment, they can generalize in the sense they know what the value of the novel options are and select them even though they have never experienced them. - How is cognitive maps/generalization related to directed vs random exploration? Not sure this is addressed in the introduction, though the authors set out to test it. - The reader would benefit from a short introduction to GPs and the motivation for using them - why are they a good model to be used here? How are they related to previous ideas mentioned in the intro? Could be very brief and since there is more in the Results.

Thank you for these excellent suggestions. We have added the following sentence to the introduction to explain that by generalization, we are referring to predictions about novel stimuli dependent on the similarity between experiences:

> These representations of distance facilitate generalization, allowing for predictions about novel stimuli based on their similarity to previous experiences.

In addition, we clarified the relationship between cognitive maps/generalization and different forms of exploration. Directed exploration is dependent on representations of uncertainty in the environment, while random exploration is not. Thus, a cognitive map and the Bayesian predictions of the GP supports directed exploration, by constructing an adaptive representation of uncertainty based on generalizations from previous observations. While your suggestion was to address this in the introduction, we felt that it would be most appropriate after we have introduced the models. Thus, we have added the following text in the discussion (also quoted in response to Section 4.7 of this letter):

> Lastly, the question of "how the cognitive map is learned" is distinct from the question of "how the cognitive map is used". Here, we have focused on the latter, and used the RBF kernel to provide a map based on the assumption of random transitions, similar to a random-policy implementation of the SR. While both the SR and GP provide a theory of how people utilize a cognitive map for performing predictive inferences, only the GP provides a theory about representations of uncertainty via Bayesian predictions of reward. These representations of uncertainty are a key feature that sets the GP apart from the SR. Psychologically, GP uncertainty estimates systematically capture participant confidence judgments and provides

the basis for uncertainty-directed exploration. This distinction may also be central to the different patterns of search we observed in spatial and non-spatial domains, where a reduction in uncertainty-directed exploration may also reflect computational differences in the structure of inference. However, the nature of these representations remains an open question for future neuroimaging research.

Lastly, to motivate our use of the GP, we added the following text to the introduction

> we used Gaussian Process (GP) regression (Rasmussen & Williams, 2006; Schulz, Speekenbrink, & Krause, 2017) as a Bayesian model of generalization *based on the principle of function learning*. [Emphasis indicates added text]

4.9 Figure suggestion

> Suggestion: Figure 1 - why not show all the gabors to illustrate the structure of the stimulus space, to show the correspondence to the spatial structure? It is not clear that this is the case. Could be nice to have the supplement figure S1 included here, if it fits.

We appreciate the suggestion for Figure 1. However, we were not able to add the entire set of Gabors due to space limitations, and without sacrificing the readability of the other panels in Figure 1. The examples of Gabor patches shown in the screenshot from Fig. 1B provides a diverse sample of the stimulus space, which we believe should be sufficient for the main text. The interested reader can find the full page figure in the first page of the supplement as Fig. S1.

References

Acuna, D., & Schrater, P. (2008). Bayesian modeling of human sequential decision-making on the multi-armed bandit problem. In *Proceedings of the 30th annual conference of the cognitive science society* (Vol. 100, pp. 200–300).

Aronov, D., Nevers, R., & Tank, D. W. (2017). Mapping of a non-spatial dimension by the hippocampal–entorhinal circuit. *Nature*, *543*(7647), 719.

Austerweil, J. L., Sanborn, S., & Griffiths, T. L. (2019). Learning how to generalize. *Cognitive science*, *43*(8).

Barr, D. J., Levy, R., Scheepers, C., & Tily, H. J. (2013). Random effects structure for confirmatory hypothesis testing: Keep it maximal. *Journal of memory and language*, *68*(3), 255–278.

Bellmund, J. L., De Cothi, W., Ruiter, T. A., Nau, M., Barry, C., & Doeller, C. F. (2020). Deforming the metric of cognitive maps distorts memory. *Nature Human Behaviour*, *4*(2), 177–188.

Bobak, C. A., Barr, P. J., & O'Malley, A. J. (2018). Estimation of an inter-rater intra-class correlation coefficient that overcomes common assumption violations in the assessment of health measurement scales. *BMC medical research methodology*, *18*(1), 93.

Bürkner, P.-C. (2017). brms: An R package for Bayesian multilevel models using Stan. *Journal of Statistical Software*, *80*(1), 1–28. doi: 10.18637/jss.v080.i01

Carroll, J. D. (1963). Functional learning: The learning of continuous functional mappings relating stimulus and response continua. *ETS Research Bulletin Series*, *1963*, i–144.

Cogliati Dezza, I., Cleeremans, A., & Alexander, W. (2019). Should we control? the interplay between cognitive control and information integration in the resolution of the exploration-exploitation dilemma. *Journal of Experimental Psychology: General*.

Collins, A. G., & Frank, M. J. (2018). Within-and across-trial dynamics of human eeg reveal cooperative interplay between reinforcement learning and working memory. *Proceedings of the National Academy of Sciences*, *115*, 2502–2507.

Constantinescu, A. O., O'Reilly, J. X., & Behrens, T. E. (2016). Organizing conceptual knowledge in humans with a gridlike code. *Science*, *352*, 1464–1468.

Dayan, P. (1993). Improving generalization for temporal difference learning: The successor representation. *Neural Computation*, *5*, 613–624.

DeLosh, E. L., Busemeyer, J. R., & McDaniel, M. A. (1997). Extrapolation: The sine qua non for abstraction in function learning. *Journal of Experimental Psychology: Learning, Memory, and Cognition*, *23*(4), 968.

Findling, C., Skvortsova, V., Dromnelle, R., Palminteri, S., & Wyart, V. (2019). Computational noise in reward-guided learning drives behavioral variability in volatile environments. *Nature neuroscience*, *22*(12), 2066–2077.

Gelman, A., & Hill, J. (2006). *Data analysis using regression and multilevel/hierarchical models*. Cambridge university press.

Gershman, S. J. (2018). Deconstructing the human algorithms for exploration. *Cognition*, *173*, 34–42.

Griffiths, T. L., Lucas, C., Williams, J., & Kalish, M. L. (2009). Modeling human function learning with gaussian processes. In *Advances in Neural Information Processing Systems* (pp. 553–560).

Hills, T. T. (2006). Animal foraging and the evolution of goal-directed cognition. *Cognitive Science*, *30*, 3–41.

Hills, T. T., Todd, P. M., & Goldstone, R. L. (2008). Search in external and internal spaces: Evidence for generalized cognitive search processes. *Psychological Science*, *19*, 802–808.

Hills, T. T., Todd, P. M., & Goldstone, R. L. (2010). The central executive as a search process: Priming exploration and exploitation across domains. *Journal of Experimental Psychology: General*, *139*(4),

590.
Hoffman, M. D., & Gelman, A. (2014). The No-U-Turn sampler: adaptively setting path lengths in Hamiltonian Monte Carlo. *Journal of Machine Learning Research*, *15*, 1593–1623.
Jäkel, F., Schölkopf, B., & Wichmann, F. A. (2008). Similarity, kernels, and the triangle inequality. *Journal of Mathematical Psychology*, *52*, 297–303.
Kwantes, P. J., & Neal, A. (2006). Why people underestimate y when extrapolating in linear functions. *Journal of Experimental Psychology: Learning, Memory, and Cognition*, *32*, 1019.
Liu, H., Ong, Y.-S., Shen, X., & Cai, J. (2020). When Gaussian process meets big data: A review of scalable GPs. *IEEE Transactions on Neural Networks and Learning Systems*.
Lucas, C. G., Griffiths, T. L., Williams, J. J., & Kalish, M. L. (2015). A rational model of function learning. *Psychonomic Bulletin & Review*, *22*(5), 1193–1215.
Machado, M. C., Rosenbaum, C., Guo, X., Liu, M., Tesauro, G., & Campbell, M. (2018). Eigenoption discovery through the deep successor representation. In *Proceedings of the International Conference on Learning Representations (ICLR).*
Mark, S., Moran, R., Parr, T., Kennerley, S., & Behrens, T. (2019). Transferring structural knowledge across cognitive maps in humans and models. *bioRxiv*.
Mehta, M. R., Quirk, M. C., & Wilson, M. A. (2000). Experience-dependent asymmetric shape of hippocampal receptive fields. *Neuron*, *25*(3), 707–715.
Mok, R. M., & Love, B. C. (2019). A non-spatial account of place and grid cells based on clustering models of concept learning. *Nature communications*, *10*(1), 1–9.
Nadel, L. (1991). The hippocampus and space revisited. *Hippocampus*, *1*(3), 221–229.
Ohl, S., & Rolfs, M. (2018). Saccadic selection of stabilized items in visuospatial working memory. *Consciousness and Cognition*, *64*, 32–44.
Radulescu, A., Niv, Y., & Ballard, I. (2019). Holistic reinforcement learning: The role of structure and attention. *Trends in Cognitive Sciences*, *23*, 278–292.
Rasmussen, C., & Williams, C. (2006). *Gaussian Processes for Machine Learning*. MIT Press.
Rigoux, L., Stephan, K. E., Friston, K. J., & Daunizeau, J. (2014). Bayesian model selection for group studies—revisited. *Neuroimage*, *84*, 971–985.
Russek, E. M., Momennejad, I., Botvinick, M. M., Gershman, S. J., & Daw, N. D. (2017). Predictive representations can link model-based reinforcement learning to model-free mechanisms. *PLoS computational biology*, *13*(9), e1005768.
Sanders, H., Wilson, M. A., & Gershman, S. J. (2019). Hippocampal remapping as hidden state inference. *BioRxiv*.
Schulz, E., Speekenbrink, M., & Krause, A. (2017). A tutorial on Gaussian process regression: Modelling, exploring, and exploiting functions. *bioRxiv*. doi: 10.1101/095190
Schulz, E., Wu, C. M., Ruggeri, A., & Meder, B. (2019). Searching for rewards like a child means less generalization and more directed exploration. *Psychological Science*. doi: 10.1177/0956797619863663
Speekenbrink, M., & Konstantinidis, E. (2015). Uncertainty and exploration in a restless bandit problem. *Topics in Cognitive Science*, *7*, 351–367.
Stachenfeld, K. L., Botvinick, M. M., & Gershman, S. J. (2017, Oct 02). The hippocampus as a predictive map. *Nature Neuroscience*, *20*, 1643 EP -. Retrieved from `http://dx.doi.org/10.1038/nn.4650` (Article)
Stephan, K. E., Penny, W. D., Daunizeau, J., Moran, R. J., & Friston, K. J. (2009). Bayesian model selection for group studies. *Neuroimage*, *46*, 1004–1017.
Sutton, R. S., & Barto, A. G. (1998). *Reinforcement learning: An introduction*. Cambridge: MIT Press.
Todd, P. M., Hills, T. T., & Robbins, T. W. (2012). *Cognitive search: Evolution, algorithms, and the*

brain. MIT press.
Whittington, J. C., Muller, T. H., Mark, S., Chen, G., Barry, C., Burgess, N., & Behrens, T. E. (2019). The tolman-eichenbaum machine: Unifying space and relational memory through generalisation in the hippocampal formation. *bioRxiv*, 770495.
Wilcox, R. R., & Rousselet, G. A. (2018). A guide to robust statistical methods in neuroscience. *Current protocols in neuroscience*, *82*(1), 8–42.
Wu, C. M., Schulz, E., Gerbaulet, K., Pleskac, T. J., & Speekenbrink, M. (2019). Under pressure: The influence of time limits on human exploration. In A. Goel, C. Seifert, & C. Freksa (Eds.), *Proceedings of the 41st Annual Conference of the Cognitive Science Society* (p. 1219—1225). Montreal, QB: Cognitive Science Society.
Wu, C. M., Schulz, E., & Gershman, S. J. (2020). Inference and search on graph-structured spaces. *bioRxiv*. doi: 10.1101/2020.01.21.981399
Wu, C. M., Schulz, E., Speekenbrink, M., Nelson, J. D., & Meder, B. (2018). Generalization guides human exploration in vast decision spaces. *Nature Human Behaviour*, *2*, 915—924. doi: 10.1038/s41562-018-0467-4
