# Supplementary material for: Similarities and differences in spatial and non-spatial cognitive maps
Source: PLoS Comput Biol. 2020 Sep 9;16(9):e1008149. doi: 10.1371/journal.pcbi.1008149 (PMC7480875; doi:10.1371/journal.pcbi.1008149)
Supplement: Attachment — Submitted filename: Review.pdf [file pcbi.1008149.s016.pdf]

Review for: *Similarities and differences in spatial and non-spatial cognitive maps*

Olivia Guest[1,2]

[1] *Research Centre on Interactive Media, Smart Systems and Emerging Technologies — RISE, Nicosia, Cyprus*
[2] *Department of Experimental Psychology, University College London, London, UK, o.guest@ucl.ac.uk*

May 12, 2020

Contents

1 Overview **1**

2 Main issues **2**
2.1 Issue A: Both domains are spatial 2
2.2 Issue B: One domain is more familiar than the other 3
2.3 Issue C: Cognitive maps are not being tested 3
2.4 Issue D: Assuming primacy of one domain 3
2.5 Issue E: Assuming Gabor task is intuitive 3
2.6 Issue F: Exploration strategies and task order 4

3 Conclusion **4**

1 Overview

I have thought deeply about this work and I hope the authors and I can communicate in a constructive way. The experiment, analysis, modelling, and code, presented in (or linked to from) the manuscript are totally fine, but I'm still stumbling over the same theoretical issues. I hope that a rethinking of the core points this manuscript currently raises is possible. I am pleased with the majority of the edits the authors have made, but I believe my main issues have not been addressed. As I said previously:

> "Overall, I am unsure about how generalisable these results are, given the authors have not sufficiently proposed any strong theoretical constraints on their hypotheses. I would have loved to have seen a higher-level theoretical account as to why they designed their experiment the way they did, why they suspected sampling strategies would differ (or perhaps they did not?), and especially why they chose the stimuli used."

That has not been addressed. This was at the core of my review but perhaps I should have explained further. I will attempt to do so in this review. One thing to keep in mind is that all my comments are core to the manuscript so much so that almost everything I mention can be seen as relating to subsets of words in the manuscript's title.

2 Main issues

Generally, there are remaining points of contention that we need to discuss. Based on the authors' response to my comments, I believe the crux of the misalignment of views on the manuscript and work generally between myself and them is down to an inference objection. Therefore, in the interests of intellectual honesty and transparency, I believe it is fair to state my views clearly: I don't agree with how the authors understand/conceptualise domains/representations of knowledge of stimuli. I don't agree that the one task is "conceptual" in terms of both what the authors want to say based on it and in terms of the embedded meaning of that work in cognitive [neuro]science. The word "conceptual" as used will most likely confuse readers coming from relevant literature that deal with conceptual representation like categorisation and semantic memory researchers.

Unfortunately, I don't believe the authors have engaged with or understood what I had to say — although I am fully open to the idea that this is down to the nature of the communication medium of the peer review process. In other words, I don't believe they are intentionally avoiding addressing my concerns but that that is indeed the results of the current revision. I also really appreciate the clarity in their write-up of their response to the reviewers, but the substance is not ideal. In this light, it's probably no surprise that I'm not currently satisfied. I don't see compelling evidence for the conclusions they draw within their own work. I will attempt to elaborate below the main issues again, as I believe they remain unaddressed. However, I think I covered a lot of the following in my previous review, so keep those words in mind as well when you read this review.

2.1 Issue A: Both domains are spatial

The authors say (response letter, page 7):

> "the purpose of our study was to ask whether there are domain-general decision making computations that rely on the similarity between stimuli, even though the particularities of how stimuli are mapped into a similarity space may differ between domains."

This is sensible to a point, but I don't think one can really test the claim of the existence of "domain-general decision making computations" with two very similar domains. That is, two domains (which both contain spatial properties, which the authors agree on, I take it, based on their response) are not enough to make strong claims about domain-generalisation. The multiple (not just two, ideally) domains tested must be different in dramatic (and specific) ways to enhance the believability of the existence of such "domain-general" mechanisms. Importantly, I don't think the evidence as presented in this manuscript makes that case compellingly, so using very confident phraseology is remiss. This is why I am happy to see that the authors have modified their text to explain that indeed the two domains are both spatial in some sense. It still worries me though, perhaps understandably, because the impression from reading the manuscript (or even just the title) is still that "domain-general" can be tested using such similar tasks.

Ultimately, the authors place Gabor patches into a 2D (or similar) space ("conceptual task") in order to compare generalisation to a 2D space ("spatial task) and this is perhaps begging the question. Gabor patches will thus appear "mapped" to a "map" similar to that of the "spatial task". There are (arguably more real, rich, amodal) concepts (e.g., cats, dogs, etc.) that can be (and have been) tested for "map" substrates (see the categorisation, conceptual learning, semantic memory, and so on, literatures) which don't have the spatial structure baked-in and which people have extensive experience with much like space (e.g., words/concepts with low age-of-acquisition). Thus avoiding perhaps begging the question.

What I discussed before and below about the keyboard arrows (*Issue E: Assuming Gabor task is intuitive*), is found in the results of their study. The authors saw that "experience with spatial search boosted performance on conceptual search, but not vice versa." (Quote from abstract and caption of figure 2.) For lack of a better phrase, this indicates to me that the "spatial task" primes one to think of Gabor patches by placing them into 2D space (which is optimal given the task as designed by the authors). This can be seen as evidence to my claims above that the task perhaps begs the question. As I mentioned above, they designed the Gabor task in a way that solving it by using a 2D mapping

makes the task easier. This is not inherently problematic, just a piece of the puzzle that needs to be stated and clarified for the sake of scientific honesty and rigour. I am glad the authors agree with me on this, from what I gather, but I feel the need to clarify given: *a*) their claims in their response letter, *b*) that the authors decided they didn't want to explore the input space for the Gabor task even though *c*) I think this (*Issue A: Both domains are spatial*) calls into question a lot of the claims including the use of ”non-spatial” in the title and throughout the manuscript.

2.2 Issue B: One domain is more familiar than the other

It is not clear to me how a domain in which people are familiar from daily life and video games (called spatial in this work) can be fairly compared to one of Gabor patches where participants are unfamiliar/unlikely to have seen and interacted with such grated patterns. I understand the authors have attempted to control for this, but I dispute that these differences can be controlled for when we're talking about years of experience in spatial movements and not in Gabor patches. The authors have stated they don't believe there are equally familiar non-spatial domains to people, but I would claim there are, e.g., auditory or phonological stimuli are good cases in point and also a much more compelling domain difference than the two visual/spatial domains used in the current work. There are many other domains to choose from either semantic or linguistic (in any form, written, spoken, etc.) or even a dramatic change in modality such as touch or olfaction. Such empirical data would provide highly compelling evidence for the authors' claims about domain-generality. So again as above, I'm a little worried about claims for domain-general mechanisms as a function of the evidence presented.

2.3 Issue C: Cognitive maps are not being tested

Another point of clarification and indeed perhaps misalignment of views revolves around the use of "cognitive maps". No evidence is given in the presented work to add to the support for a hippocampal-entorhinal "cognitive map" used in the tasks, for example, even though this is prominently stated in the abstract of the manuscript. I don't dispute that a hippocampal-entorhinal "cognitive map" might be recruited to carry out these tasks (bear in mind my disagreement is an inference objection), I'm just unsure why this is so prominently placed given this is a behavioural experiment. Besides, is a map being used because the task requires one or primes one or is a map being used because that is how people solve ”conceptual” tasks? We can't know for certain from the current study — but we can know for certain no direct evidence for a hippocampal-entorhinal "cognitive map" is being presented.

2.4 Issue D: Assuming primacy of one domain

The authors make a very strong claim (in their response letter, page 10) that I think belies their strong assumptions (and, perhaps inadvertently, hinting at a lack of interest in probing them) in their work generally:

> "No domain is more central to the human experience than the spatial world around us. [...] Thus, it might be an impractical or perhaps quixotic endeavor to seek out conceptual stimuli that are equally familiar as any spatial stimuli"

I am not sure I am comfortable with this. It could easily be argued that what is central to the human experience is our vast linguistic capacity and not that we move through or understand space. I'm not sure why it's needed for this research to make such a strong untested claim. Claiming that "the spatial role around us" is "central" also serves to highlight how the authors are perhaps begging the question since they assert the primacy of one domain even though this aspect is what they want to test: domain-generalisation. For more on how they can address this see especially *Issue A: Both domains are spatial* and *Issue B: One domain is more familiar than the other.*

2.5 Issue E: Assuming Gabor task is intuitive

Another very strong claim the authors make in their response is that up, down, left, right, etc., are somehow indubitably and consistently mapped to specific concepts that are coherent with some ideal.

The authors say (in their response letter, page 11):

> "Thus, while it is certainly intuitive to move through a 2D space with arrow keys, we also believe that the input space for our Gabor stimuli is similarly intuitive."

I disagree and find this a bit bizarre. It is a very strong claim without any evidence. Indeed counter-evidence is provided in their own results since we see a facilitation effect if the Gabor task is done after the "spatial task". Ergo the Gabor task not "similalrly intuitive" to the spatial one. From page 9 of the manuscript:

> "Participants were boosted by a one-directional transfer effect, where experience with the spatial task improved performance on the conceptual task, but not the other way around."

To add an anthropological take, in one of my native languages nodding upwards means "no" and downwards "yes". Symbols (including arrows and directionality) mean things given a context. In an anglosphere setting somebody nodding up (and down) will mean "yes", even to me. To wit, the meaning of the arrow keys of the keyboard are specifically learned as part of 2/3D video games, as part of the given task, etc., and will carry with them context-consistent baggage. So in the current task, participants are primed (for lack of a better word) by what arrow keys (not arrows generally, but arrow keys) normally mean (and have meant in other similar/previous tasks): movement in space. As mentioned, arrows generally (not just on the keyboard) are imbibed with meaning primarily due to context. My comment on the arrow keys of the keyboard was to highlight that in the context of the task, they are likely spatially mapped. This is something we agree on, I hope/think, but deserved clarification given the issues I mention in this section and *Issue A: Both domains are spatial*.

2.6 Issue F: Exploration strategies and task order

From page 11 of the manuscript:

> "[P]articipants displayed similar and somewhat correlated levels of generalization in both tasks, but with markedly different patterns of exploration. Whereas participants engaged in typical levels of directed exploration in the spatial domain [,] they displayed reduced levels of directed exploration in the conceptual task, substituting instead an increase in undirected exploration. [T]his indicates a fundamental difference in how people represent or reason about spatial and conceptual domains in order to decide which are the most promising options to explore."

Firstly, this is not a fundamental difference given the evidence. It could be. But it could also be a side-effect of less experience in that domain or less appropriate "mapping" of the Gabor patches onto the more useful strategy as described above of mapping them onto a 2D space. I mentioned this in my previous review too and I assume the authors agree but forgot to amend their text.

Secondly and importantly, do participants show different exploration strategies in the "conceptual task" as a function of task order? This should be very useful and relevant to discuss or at least mention in the current paper either way. It's a direct repercussion of my idea of spatial priming above (*Issue E: Assuming Gabor task is intuitive*). This is why I mentioned previously the idea of making a theoretical/conceptual model of the "maps" — documenting (hopefully formally) the experimenters' assumptions of how these theoretical entities interact — and how domains might access a domain-general "map" is useful: one can make clear falsifiable generalisable replicable conclusions and even predictions (Guest & Martin, 2020).

3 Conclusion

I propose one way to resolve this is that the authors can reconsider their chosen framing of the work. This is what I have been hoping for all along: a deep reevaluation of the core points this manuscript is touching on. The experiment itself is fine, but it cannot be interpreted as an investigation into "domain-generalisation" when it's possible that that is not what is being tested — or into a contrast

between two different domains when they are not that different — or to be about maps when I'm not convinced the maps are as described/implied, and so on (refer to everything I say above). These are issues about concepts central to the manuscript — so much so that some of them they are in the title.

Ultimately, I don't believe their results are supportive to their arguments as currently presented in their manuscript (see all my above comments and previous review). The text should reflect this by more than a few changes of phrase. Ideally, they should: formalise their claims (even if eventually found to be unsupported by the evidence), demonstrate why they ran the experiment the way they did given these claims, think about how the results do or don't give credence to their beliefs (about Gabor patches, for example), and address the issues presented in this review.

It is highly possible that these issues are a function of a lack of a common framework (Guest & Martin, 2020). If so, may I remind them of my previous review: "the authors have not sufficiently proposed any strong theoretical constraints on their hypotheses." Given a lot of the core of what I raised in my previous review (as demonstrated above) remains unaddressed, I cannot currently recommend acceptance. Notwithstanding, I believe/hope the authors agree with me on this meta-issue and will strive in good faith to amend their manuscript.

References

Guest, O., & Martin, A. E. (2020). How computational modeling can force theory building in psychological science.
doi: 10.31234/osf.io/rybh9
