# Supplementary material for: Similarities and differences in spatial and non-spatial cognitive maps
Source: PLoS Comput Biol. 2020 Sep 9;16(9):e1008149. doi: 10.1371/journal.pcbi.1008149 (PMC7480875; doi:10.1371/journal.pcbi.1008149)
Supplement: Attachment — Submitted filename: reviewerRebuttal2ndresubmission.pdf [file pcbi.1008149.s017.pdf]

Contents

1 Reviewer 1 **3**

1.1 Overview . . . 3
1.2 Main issues . . . 3
1.3 Issue A: Both domains are spatial . . . 4
1.4 Issue B: One domain is more familiar than the other . . . 6
1.5 Issue C: Cognitive maps are not being tested . . . 7
1.6 Issue D: Assuming primacy of one domain . . . 8
1.7 Issue E: Assuming Gabor task is intuitive . . . 9
1.8 Issue F: Exploration strategies and task order . . . 10
1.9 Conclusion . . . 12

1 Reviewer 1

1.1 Overview

> I am pleased with the majority of the edits the authors have made, but I believe my main issues have not been addressed. As I said previously: "Overall, I am unsure about how generalisable these results are, given the authors have not sufficiently proposed any strong theoretical constraints on their hypotheses. I would have loved to have seen a higher-level theoretical account as to why they designed their experiment the way they did, why they suspected sampling strategies would differ (or perhaps they did not?), and especially why they chose the stimuli used."

We are glad the reviewer is pleased with the majority of our edits, and we are grateful for the generous time and effort put into these reviews. Particularly, we appreciate the depth with which some of the issues from the previous round of reviews have been unpacked and discussed in detail in the latest round of feedback. Below, we discuss our response to these arguments and document our changes to the manuscript in response to this feedback. We believe this feedback has contributed to a better version of our paper.

1.2 Main issues

> Generally, there are remaining points of contention that we need to discuss. Based on the authors' response to my comments, I believe the crux of the misalignment of views on the manuscript and work generally between myself and them is down to an inference objection. Therefore, in the interests of intellectual honesty and transparency, I believe it is fair to state my views clearly: I don't agree with how the authors understand/conceptualise domains/representations of knowledge of stimuli. I don't agree that the one task is "conceptual" in terms of both what the authors want to say based on it and in terms of the embedded meaning of that work in cognitive [neuro]science. The word "conceptual" as used will most likely confuse readers coming from relevant literature that deal with conceptual representation like categorisation and semantic memory researchers.
>
> Unfortunately, I don't believe the authors have engaged with or understood what I had to say – although I am fully open to the idea that this is down to the nature of the communication medium of the peer review process. In other words, I don't believe they are intentionally avoiding addressing my concerns but that that is indeed the results of the current revision. I also really appreciate the clarity in their write-up of their response to the reviewers, but the substance is not ideal. In this light, it's probably no surprise that I'm not currently satisfied. I don't see compelling evidence for the conclusions they draw within their own work. I will attempt to elaborate below the main issues again, as I believe they remain unaddressed. However, I think I covered a lot of the following in my previous review, so keep those words in mind as well when you read this review

We thank the reviewer for providing such a clear description of the central point of contention. Specifically, the argument presented here is that our task using Gabor patch stimuli should not be considered "conceptual" in the context of cognitive [neuro]science.

While there are some contexts in which we would fully agree (e.g., cognitive linguistics; Lakoff & Johnson, 2008), we believe that specific to the topic of decision-making and reward learning in cognitive neuroscience and cognitive science, our use of the term "conceptual" is both justified and beneficial for connecting to related literature on the topic. For instance, the work of Constantinescu, O'Reilly, and

Behrens (2016), who in their paper "Organizing conceptual knowledge in humans with a gridlike code" used abstract bird stimuli varying along two dimensions (leg length and neck length) as a form of conceptual knowledge. In addition, Theves, Fernandez, and Doeller (2019) uses circle stimuli that varied along two "conceptual" dimensions (size and opacity) in a task for studying "conceptual knowledge" and "concept learning". Similarly, the study of concept learning in psychology has commonly relied on novel, abstract stimuli (Kumaran, Summerfield, Hassabis, & Maguire, 2009; Mack, Love, & Preston, 2016), often with spatial properties (Austerweil & Griffiths, 2010; Austerweil, Sanborn, & Griffiths, 2019; Shepard, Hovland, & Jenkins, 1961; Tenenbaum, 1999). Thus, our Gabor stimuli contains spatial properties (Issue A), were novel to participants (Issue B), and less intuitive than an analogous spatial task (Issue E) — not distinct from — but in common with a large set of conceptual learning stimuli used in cognitive science/neuroscience.

In his seminal book *Conceptual Spaces: the Geometry of Thought*, Gärdenfors (2004) defines conceptual spaces as being "built upon geometrical structures based on a number of quality dimensions", such as temperature, weight, brightness, pitch, height, width, and depth. The fundamental role of these *quality dimensions* is that they "make it possible to talk about distances along the dimensions". The tilt and stripe frequency dimensions of our Gabor patch stimuli are the quality dimensions of our conceptual task, which fulfill the role of allowing both participants and computational models to describe distances. The reviewer is reluctant to agree that our stimuli should be considered "conceptual", which is understandable, since as Medin and Rips (2005) put it, "[t]he concept of concepts is difficult to define". To this end, we have amended the following section of our General Discussion to clarify that i) there are a wide range of alternative non-spatial stimuli we have not tested, ii) other stimuli might be considered "more conceptual", and iii) we make no claims about which other domains may also be described using the same framework.

> There is also a wide range of alternative non-spatial stimuli that we have not tested (for instance auditory (Aronov, Nevers, & Tank, 2017) or linguistic stimuli (Abbott, Austerweil, & Griffiths, 2015; Hills, Jones, & Todd, 2012)), which could be considered more "conceptual" than our Gabor stimuli or may be more familiar to participants. Thus, it is an open empirical question to determine the limits to which spatial and different kinds of conceptual stimuli can be described using the same computational framework.

In the following sections, we respond to each of the individual issues raised in this round of reviews.

1.3 Issue A: Both domains are spatial

> The authors say (response letter, page 7): "the purpose of our study was to ask whether there are domain-general decision making computations that rely on the similarity between stimuli, even though the particularities of how stimuli are mapped into a similarity space may differ between domains."
>
> This is sensible to a point, but I don't think one can really test the claim of the existence of "domain-general decision making computation" with two very similar domains. That is, two domains (which both contain spatial properties, which the authors agree on, I take it, based on their response) are not enough to make strong claims about domain-generalisation. The multiple (not just two, ideally) domains tested must be different in dramatic (and specific) ways to enhance the believability of the existence of such "domain-general" mechanisms. Importantly, I don't think the evidence as presented in this manuscript makes that case compellingly, so using very confident phraseology is remiss. This is why I am happy to see that the authors have modified their text to explain that indeed the two domains are both spatial

in some sense. It still worries me though, perhaps understandably, because the impression from reading the manuscript (or even just the title) is still that "domain-general" can be tested using such similar tasks.

Ultimately, the authors place Gabor patches into a 2D (or similar) space ("conceptual task") in order to compare generalisation to a 2D space ("spatial task") and this is perhaps begging the question. Gabor patches will thus appear "mapped" to a "map" similar to that of the "spatial task". There are (arguably more real, rich, amodal) concepts (e.g., cats, dogs, etc.) that can be (and have been) tested for "map" substrates (see the categorisation, conceptual learning, semantic memory, and so on, literatures) which don't have the spatial structure baked-in and which people have extensive experience with much like space (e.g., words/concepts with low age-of-acquisition). Thus avoiding perhaps begging the question.

What I discussed before and below about the keyboard arrows (Issue E: Assuming Gabor task is intuitive), is found in the results of their study. The authors saw that "experience with spatial search boosted performance on conceptual search, but not vice versa." (Quote from abstract and caption of figure 2.) For lack of a better phrase, this indicates to me that the "spatial task" primes one to think of Gabor patches by placing them into 2D space (which is optimal given the task as designed by the authors). This can be seen as evidence to my claims above that the task perhaps begs the question. As I mentioned above, they designed the Gabor task in a way that solving it by using a 2D mapping makes the task easier. This is not inherently problematic, just a piece of the puzzle that needs to be stated and clarified for the sake of scientific honesty and rigour. I am glad the authors agree with me on this, from what I gather, but I feel the need to clarify given: a) their claims in their response letter, b) that the authors decided they didn't want to explore the input space for the Gabor task even though c) I think this (Issue A: Both domains are spatial) calls into question a lot of the claims including the use of "non-spatial" in the title and throughout the manuscript.

The argument presented here is that both domains we tested are spatial, and that one cannot test for domain-general decision making computations with two very similar domains.

First of all, we would like to clarify that the term "domain-general" was only used in the previous rebuttal letter, and is found nowhere in the manuscript. We agree that this term was used imprecisely in our rebuttal letter, and we apologize for the confusion this may have caused. In particular, the reviewer seems to suggest that what we were trying to study "domain-generalization", which could be interpreted as a form of remapping from one domain to another or as a form of generalization across domains. We apologize for this misunderstanding, and have revised our introduction to clarify that the goal of our paper is to test whether the same principles of generalization and exploration apply in the two specific domains we test:

> Here, we ask to what extent does the search for rewards depend on the same distance-dependent generalization across *two different domains — one defined by spatial location and another by abstract features of a Gabor patch — despite potential differences in how the stimuli and their similarities may be processed?* [emphasis added to highlight changes to text]

Now, the main issue we need to address is whether our Gabor task is too similar to our spatial task to measure meaningful differences. We agree that the tasks are similar in that they both map onto the same motor inputs (button presses), which gives the Gabor task a spatial element based on the spatial relationship between buttons. Thus, it is possible that generalization and search occurs over this shared

motor representation (with spatial qualities), rather than the perceptual stimulus dimensions. However, this is unlikely to be the case, because we found statistically reliable changes in behavior and in our models, across tasks. This suggests that the computations involved in the Gabor task likely occurred over the stimulus features rather than the motor inputs.

While the reviewer makes some suggestions about alternative stimuli to use, such as amodal concepts of cats and dogs (perhaps inspired by Freedman, Riesenhuber, Poggio, & Miller, 2001), we had previously considered and subsequently rejected this exact class of stimuli[1]. Our primary motivation when selecting stimuli was to keep the computational description as equivalent as possible across tasks, in order to equitably compare behavior. As a starting point, we chose to map our Gabor stimuli to a 2D feature space, to mirror previous work by Constantinescu et al. (2016). This allowed us to investigate the downstream behavioral implications of their findings, specifically, that spatial and conceptual stimuli might share the similar map-like representations. Nowhere in our paper nor in Constantinescu et al. (2016) is the claim made that it is *necessarily* the case that all forms of conceptual stimuli share a representation with an analogous spatial domain. Rather, we take as a premise that both domains can share the same representation (based on previous neuroimaging research; Aronov et al., 2017; Constantinescu et al., 2016; Garvert, Dolan, & Behrens, 2017; Schuck, Cai, Wilson, & Niv, 2016; Solomon, Lega, Sperling, & Kahana, 2019). This necessitated that we stick closely to a choice of conceptual stimuli that was as commensurate as possible to an analogous spatial task. For this purpose, a novel, abstract stimulus set mapped to a 2D feature space (mirroring the stretchy bird stimuli set) was the clear choice.

Thus, our primary concern during the design of our experiment was in the opposite direction from the issue raised here. We wanted to avoid designing a conceptual task that would be too different from our spatial task to facilitate a fair comparison. However, it remains an open scientific question to discover the exact boundaries, where one could hypothesize that tasks of lesser difference would cease to produce differences in exploration, or that tasks of greater difference would cease to produce similar patterns of generalization. We have made changes to the General Discussion to address other possible domains:

> There is also a wide range of alternative non-spatial stimuli that we have not tested (for instance auditory (Aronov et al., 2017) or linguistic stimuli (Abbott et al., 2015; Hills et al., 2012)), which could be considered more "conceptual" than our Gabor stimuli or may be more familiar to participants. Thus, it is an open empirical question to determine the limits to which spatial and conceptual stimuli can be described using the same computational framework.

1.4 Issue B: One domain is more familiar than the other

> It is not clear to me how a domain in which people are familiar from daily life and video games (called spatial in this work) can be fairly compared to one of Gabor patches where participants are unfamiliar/unlikely to have seen and interacted with such grated patterns. I understand the authors have attempted to control for this, but I dispute that these differences can be controlled for when we're talking about years of experience in spatial movements and not in Gabor patches. The authors have stated they don't believe there are equally familiar non-spatial domains to people, but I would claim there are, e.g., auditory or phonological stimuli are good cases in point and also a much more compelling domain difference than the two visual/spatial domains used in the current work. There are many other domains to choose from either semantic or linguistic (in any form, written, spoken, etc.) or even a dramatic

[1]In brief: i) continuous morphs of cats and dogs suffer from the same unidentifiability issues as the stretchy birds when mapped to the large search space we required, ii) human participants tend to have strong preferences for cats vs. dogs, which could bias search decisions, iii) there are uncanny valley issues where certain areas of the stimulus space are perceived as more natural vs. unnatural due to real-world examples being non-uniformly distributed.

> change in modality such as touch or olfaction. Such empirical data would provide highly compelling evidence for the authors' claims about domain-generality. So again as above, I'm a little worried about claims for domain-general mechanisms as a function of the evidence presented.

We agree that there are differences in familiarity between domains, which is what motivated us to include an extensive training phase in order to minimize this difference. We also think that it would be very fascinating to establish a link between spatial stimuli and domains with alternative modalities, such as auditory stimuli (for instance, equivalencies shown by Aronov et al., 2017). However, that is not our goal here. Rather, we aim to test the behavioral consequences of previous claims about the equivalency between the organization of knowledge across spatial and conceptual domains (as defined in terms of a novel, abstract stimuli set mapped to a 2D feature space; Constantinescu et al., 2016). Our contribution is in understanding the downstream implications for behavior, where we are able to provide a more concrete understanding of the similarities in reward generalization and differences in patterns of exploration. We would also like to point out again that nowhere in the manuscript do we make any claims about domain-general mechanisms.

To address alternative domains that should be researched in future investigations, we have added the following text to the General Discussion (also quoted in response to Issue B):

> There is also a wide range of alternative non-spatial stimuli that we have not tested (for instance auditory (Aronov et al., 2017) or linguistic stimuli (Abbott et al., 2015; Hills et al., 2012)), which could be considered more "conceptual" than our Gabor stimuli or may be more *familiar* to participants. Thus, it is an open empirical question to determine the limits to which spatial and conceptual stimuli can be described using the same computational framework. [emphasis added]

1.5 Issue C: Cognitive maps are not being tested

> Another point of clarification and indeed perhaps misalignment of views revolves around the use of "cognitive maps". No evidence is given in the presented work to add to the support for a hippocampal-entorhinal "cognitive map" used in the tasks, for example, even though this is prominently stated in the abstract of the manuscript. I don't dispute that a hippocampal-entorhinal "cognitive map" might be recruited to carry out these tasks (bear in mind my disagreement is an inference objection), I'm just unsure why this is so prominently placed given this is a behavioural experiment. Besides, is a map being used because the task requires one or primes one or is a map being used because that is how people solve "conceptual" tasks? We can't know for certain from the current study — but we can know for certain no direct evidence for a hippocampal-entorhinal "cognitive map" is being presented.

We thank the reviewer for raising this point. The reviewer is of course correct that we are not studying the neural implementation of the cognitive map in the hippocampus/entorhinal cortex. Our aim was to study the "cognitive" side of cognitive maps, which we conceptualize as the mental representation of relative similarities between the stimuli/bandits, in line with the original work from Tolman (1948). Although our work is purely behavioral, we do believe that it has relevance for the current discussion about the neural implementation of cognitive maps (Aronov et al., 2017; Constantinescu et al., 2016; Garvert et al., 2017; Schuck et al., 2016; Solomon et al., 2019), and that the relationships between stimuli in our task may be organized in a hippocampal-entorhinal map.

In response to this feedback, we have removed any misleading phrasing and have toned down any reference to the neural implementation of this map in the paper, for instance, by removing "entorhinal-hippocampal" from the first sentence of the abstract:

> Learning and generalization in spatial domains is often thought to rely on a "cognitive map", representing relationships between spatial locations.

In addition, we have also changed the text to clarify the logical structure of our paper:

> We formalize a computational model that incorporates distance-dependent generalization and test it in a within-subject experiment, where either spatial features or abstract conceptual features are predictive of rewards. *This allows us to study the extent to which the same organizational structure of cognitive representations is used in both domains, based on examining the downstream behavioral implications for learning, decision making, and exploration.* [Emphasis added to indicate modified text]

1.6 Issue D: Assuming primacy of one domain

> The authors make a very strong claim (in their response letter, page 10) that I think belies their strong assumptions (and, perhaps inadvertently, hinting at a lack of interest in probing them) in their work generally:
>
> "No domain is more central to the human experience than the spatial world around us. [...] Thus, it might be an impractical or perhaps quixotic endeavor to seek out conceptual stimuli that are equally familiar as any spatial stimuli"
>
> I am not sure I am comfortable with this. It could easily be argued that what is central to the human experience is our vast linguistic capacity and not that we move through or understand space. I'm not sure why it's needed for this research to make such a strong untested claim. Claiming that "the spatial role around us" is "central" also serves to highlight how the authors are perhaps begging the question since they assert the primacy of one domain even though this aspect is what they want to test: domain-generalisation. For more on how they can address this see especially Issue A: Both domains are spatial and Issue B: One domain is more familiar than the other

The reviewer raises an objection to an argument we made in our previous rebuttal letter (but not present in the paper), where we questioned if it was at all possible to find conceptual stimuli that are equally familiar as spatial stimuli. Instead, the counter argument is raised that "what is central to the human experience is our vast linguistic capacity and not that we move through or understand space".

We agree that it is not within the scope of our paper to make any claims about whether one domain is more central than others. We have removed the only mention of this claim in the manuscript, where we had previously made the relatively weak conjecture that "there may be something special or central about spatial encoding Nadel (1991)". The revised sentence in the General Discussion now looks like the following:

> Thus, while both spatial and conceptual knowledge are capable of being organized into a common map-like representation, there may be domain differences in terms of the ease of learning such a map and asymmetries in the transfer of knowledge.

We also want to clarify that we are not trying to test "domain-generalisation". This seems to refer to our use of the term "domain-general decision making computations", which appeared in the previous rebuttal letter, but not in the manuscript. We addressed this point in Issue A (Section 1.3), where we acknowledge it was used imprecisely and amended the text to clarify we are not making domain general claims.

1.7 Issue E: Assuming Gabor task is intuitive

> The authors say (in their response letter, page 11):
>
> "Thus, while it is certainly intuitive to move through a 2D space with arrow keys, we also believe that the input space for our Gabor stimuli is similarly intuitive."
>
> I disagree and find this a bit bizarre. It is a very strong claim without any evidence. Indeed counterevidence is provided in their own results since we see a facilitation effect if the Gabor task is done after the "spatial task". Ergo the Gabor task not "similarly intuitive" to the spatial one. From page 9 of the manuscript:
>
> "Participants were boosted by a one-directional transfer effect, where experience with the spatial task improved performance on the conceptual task, but not the other way around."
>
> To add an anthropological take, in one of my native languages nodding upwards means "no" and downwards "yes". Symbols (including arrows and directionality) mean things given a context. In an anglosphere setting somebody nodding up (and down) will mean "yes", even to me. To wit, the meaning of the arrow keys of the keyboard are specifically learned as part of 2/3D video games, as part of the given task, etc., and will carry with them context-consistent baggage. So in the current task, participants are primed (for lack of a better word) by what arrow keys (not arrows generally, but arrow keys) normally mean (and have meant in other similar/previous tasks): movement in space. As mentioned, arrows generally (not just on the keyboard) are imbibed with meaning primarily due to context. My comment on the arrow keys of the keyboard was to highlight that in the context of the task, they are likely spatially mapped. This is something we agree on, I hope/think, but deserved clarification given the issues I mention in this section and Issue A: Both domains are spatial.

The reviewer makes the argument that the Gabor patch stimuli is not as intuitive as the spatial task, and disagrees with a comment made in our previous rebuttal letter describing the intuitiveness of the two tasks as being "similar".

We agree that our claim from the previous response letter about Gabor stimuli being "similarly inutitive" was too strong. However, in the manuscript, we clearly acknowledge the existence of differences in intuitiveness:

> [...] the arrow key inputs may have been more intuitive for manipulating the spatial stimuli. While generalization could be observed in both situations, directed exploration might require more explicitly accessible information about structural relationships or be facilitated by more intuitively mappable inputs.

While we agree that this remains a potential dynamic in our study, we have clearly identified it as such, and have designed our study to mitigate the influence as much as possible. Although it is unavoidable that any button will have a spatial relationship to any other button, the mapping of keys in the Gabor task (up/down mapped to higher or lower stripe frequency and left/right mapped to tilt in the respective directions) was designed to avoid any counter-intuition (e.g., up as reducing stripe frequency). In addition,

we employed an extensive training phase with a strict learning criterion to reduce familiarity or intuition differences. In summary, we acknowledge differences in intuition and have clearly identified this dynamic in the paper.

1.8 Issue F: Exploration strategies and task order

> From page 11 of the manuscript:
>
> "[P]articipants displayed similar and somewhat correlated levels of generalization in both tasks, but with markedly different patterns of exploration. Whereas participants engaged in typical levels of directed exploration in the spatial domain [,] they displayed reduced levels of directed exploration in the conceptual task, substituting instead an increase in undirected exploration. [T]his indicates a fundamental difference in how people represent or reason about spatial and conceptual domains in order to decide which are the most promising options to explore."
>
> Firstly, this is not a fundamental difference given the evidence. It could be. But it could also be a side-effect of less experience in that domain or less appropriate "mapping" of the Gabor patches onto the more useful strategy as described above of mapping them onto a 2D space. I mentioned this in my previous review too and I assume the authors agree but forgot to amend their text. Secondly and importantly, do participants show different exploration strategies in the "conceptual task" as a function of task order? This should be very useful and relevant to discuss or at least mention in the current paper either way. It's a direct repercussion of my idea of spatial priming above (Issue E: Assuming Gabor task is intuitive). This is why I mentioned previously the idea of making a theoretical/conceptual model of the "maps" — documenting (hopefully formally) the experimenters' assumptions of how these theoretical entities interact — and how domains might access a domain-general "map" is useful: one can make clear falsifiable generalisable replicable conclusions and even predictions (Guest & Martin, 2020).

There appear to be three parts to this issue, which we address separately.

1.8.1 Fundamental differences

We would first like to mention that in response to the previous round of reviews, we amended the term "fundamental differences" to "meaningful differences" (as quoted in Section 2.4 of the previous rebuttal letter), but due to a technical error, the change was inadvertently reverted in the submitted manuscript. We apologize for this mistake, and thank the reviewer for catching it. It has now been corrected. We quote the text below, and include the preceding sentences to add important context:

> Whereas participants engaged in typical levels of directed exploration in the spatial domain (replicating previous studies; Schulz, Wu, Ruggeri, & Meder, 2019; Wu, Schulz, Speekenbrink, Nelson, & Meder, 2018), they displayed reduced levels of directed exploration in the conceptual task, substituting instead an increase in undirected exploration. Again, this is not due to a lack of effort, because participants made longer search trajectories in the conceptual domain (see Fig. S4a). Rather, this indicates a *meaningful* difference in how people represent or reason about spatial and conceptual domains in order to decide which are the most promising options to explore. [emphasis added to indicate changed text]

Specifically, the "meaningful difference" in exploration behavior we are describing here refers to what we cannot ascribe to lack of effort on behalf of participants. The evidence for this claim is on the basis that participants had longer search trajectories in the conceptual task. We believe that this change in terminology assuages any concerns, since the preceding text provides the exact context to which we describe this difference as being meaningful (as opposed to accidental, by lack of effort).

1.8.2 Influence of task order on parameter estimates

We thank the reviewer for the suggestion to look at whether differences in β (exploration bonus) and τ (softmax temperature) estimates between tasks were also influenced by task-order. We first computed $\Delta_\beta = \beta_{\text{spatial}} - \beta_{\text{conceptual}}$ and $\Delta_\tau = \tau_{\text{spatial}} - \tau_{\text{conceptual}}$ for each participant, and then conducted a two-way ANOVA using task order and environment type to predict either Δ_β and Δ_τ. In all cases, we found no influence of task order, environment, or their interaction on differences in β or τ. We have added the following text to our results:

> These domain-specific differences in β and τ were not influenced by task order or environment (two-way ANOVA: all $p > .05$, $BF < 1$).

For transparency, we include the full set of test statistics in this rebuttal letter, which are also found in the online supplement (https://charleywu.github.io/cognitivemaps/modelingResultsNotebook.html#task-order-and-differences-in-exploration):

Δ_β	**Df**	**Sum Sq**	**F value**	**Pr(>F)**	**BF**
Environment	1	1.145	0.105	.746	0.198
TaskOrder	1	27.423	2.519	.115	0.562
Environment:TaskOrder	1	2.037	0.187	.666	0.120
Residuals	125	1360.640			

Δ_τ	**Df**	**Sum Sq**	**F value**	**Pr(>F)**	**BF**
Environment	1	82.398	1.321	.253	0.342
TaskOrder	1	86.730	1.391	.240	0.310
Environment:TaskOrder	1	91.256	1.464	.229	0.058
Residuals	125	7794.108			

1.8.3 Modeling the cognitive maps

Lastly, to address the idea of making a model of the "maps", this is exactly the role of the RBF kernel in our model, which has clear "falsifiable generalisable replicable conclusions and even predictions". The central idea behind cognitive maps is a representation of similarity, for which we use RBF kernel as a specific model for representing similarities between stimuli. We address our RBF model of the maps in relation to alternative models (e.g., successor representation) in the "Related Work" section of the General Discussion, where the following text makes explicit the assumptions that our model is making:

> Lastly, the question of "how the cognitive map is learned" is distinct from the question of "how the cognitive map is used". Here, we have focused on the latter, and used the RBF kernel to provide a map based on the assumption of random transitions, similar to a random-policy implementation of the SR. [SR: successor representation]

1.9 Conclusion

> I propose one way to resolve this is that the authors can reconsider their chosen framing of the work. This is what I have been hoping for all along: a deep reevaluation of the core points this manuscript is touching on. The experiment itself is fine, but it cannot be interpreted as an investigation into "domain-generalisation" when it's possible that that is not what is being tested — or into a contrast between two different domains when they are not that different — or to be about maps when I'm not convinced the maps are as described/implied, and so on (refer to everything I say above). These are issues about concepts central to the manuscript — so much so that some of them they are in the title.
>
> Ultimately, I don't believe their results are supportive to their arguments as currently presented in their manuscript (see all my above comments and previous review). The text should reflect this by more than a few changes of phrase. Ideally, they should: formalise their claims (even if eventually found to be unsupported by the evidence), demonstrate why they ran the experiment the way they did given these claims, think about how the results do or don't give credence to their beliefs (about Gabor patches, for example), and address the issues presented in this review.
>
> It is highly possible that these issues are a function of a lack of a common framework (Guest & Martin, 2020). If so, may I remind them of my previous review: "the authors have not sufficiently proposed any strong theoretical constraints on their hypotheses." Given a lot of the core of what I raised in my previous review (as demonstrated above) remains unaddressed, I cannot currently recommend acceptance. Notwithstanding, I believe/hope the authors agree with me on this meta-issue and will strive in good faith to amend their manuscript..

We thank the reviewer for the generous level of detail given in these comments. Across all six issues raised in the current round of reviews, and the comments made in the previous round, we have responded in depth and made substantial changes to the theoretical framing of our paper. The primary issue appears to be that the reviewer believes we are making claims about "domain-generalisation". We apologize for causing confusion by using the term in the first rebuttal letter, and have tried our best to clarify the high-level theoretical account of the paper. We hope our revision will help to avoid further misunderstandings about the scope and logical structure of our conclusions.

In response to the six individual issues issues, we have amended the text in numerous areas. To summarize, we have i) clarified that the scope of our analyses are specific to a comparison of two domains, ii) discussed alternative non-spatial stimuli, iii) conceded that other stimuli could be considered more conceptual or more intuitive than our Gabor stimuli, iv) provided a clearer formalization of the logical structure of our arguments, v) toned down mention of the neural implementation of the cognitive map, and vi) added a new analysis looking at the influence of task order on changes in exploration. We believe this has substantially improved the theoretical precision of this paper.

References

Abbott, J. T., Austerweil, J. L., & Griffiths, T. L. (2015). Random walks on semantic networks can resemble optimal foraging. *Psychological Review*, *122*(3), 558–569.

Aronov, D., Nevers, R., & Tank, D. W. (2017). Mapping of a non-spatial dimension by the hippocampal–entorhinal circuit. *Nature*, *543*(7647), 719.

Austerweil, J. L., & Griffiths, T. (2010). Learning hypothesis spaces and dimensions through concept learning. In *Proceedings of the annual meeting of the cognitive science society* (Vol. 32).

Austerweil, J. L., Sanborn, S., & Griffiths, T. L. (2019). Learning how to generalize. *Cognitive science*, *43*(8).

Constantinescu, A. O., O'Reilly, J. X., & Behrens, T. E. (2016). Organizing conceptual knowledge in humans with a gridlike code. *Science*, *352*, 1464–1468.

Freedman, D. J., Riesenhuber, M., Poggio, T., & Miller, E. K. (2001). Categorical representation of visual stimuli in the primate prefrontal cortex. *Science*, *291*(5502), 312–316.

Gärdenfors, P. (2004). *Conceptual spaces: The geometry of thought*. MIT press.

Garvert, M. M., Dolan, R. J., & Behrens, T. E. (2017). A map of abstract relational knowledge in the human hippocampal–entorhinal cortex. *eLife*, *6*, e17086.

Hills, T. T., Jones, M. N., & Todd, P. M. (2012). Optimal foraging in semantic memory. *Psychological review*, *119*(2), 431.

Kahnt, T., & Tobler, P. N. (2016). Dopamine regulates stimulus generalization in the human hippocampus. *Elife*, *5*, e12678.

Kumaran, D., Summerfield, J. J., Hassabis, D., & Maguire, E. A. (2009). Tracking the emergence of conceptual knowledge during human decision making. *Neuron*, *63*(6), 889–901.

Lakoff, G., & Johnson, M. (2008). *Metaphors We Live By*. University of Chicago press.

Mack, M. L., Love, B. C., & Preston, A. R. (2016). Dynamic updating of hippocampal object representations reflects new conceptual knowledge. *Proceedings of the National Academy of Sciences*, *113*(46), 13203–13208.

Medin, D. L., & Rips, L. J. (2005). Concepts and categories: Memory, meaning, and metaphysics. *The Cambridge handbook of thinking and reasoning*, 37–72.

Nadel, L. (1991). The hippocampus and space revisited. *Hippocampus*, *1*(3), 221–229.

Schuck, N. W., Cai, M. B., Wilson, R. C., & Niv, Y. (2016). Human orbitofrontal cortex represents a cognitive map of state space. *Neuron*, *91*, 1402–1412.

Schulz, E., Wu, C. M., Ruggeri, A., & Meder, B. (2019). Searching for rewards like a child means less generalization and more directed exploration. *Psychological Science*. doi: 10.1177/0956797619863663

Shepard, R. N., Hovland, C. I., & Jenkins, H. M. (1961). Learning and memorization of classifications. *Psychological monographs: General and applied*, *75*(13), 1.

Solomon, E. A., Lega, B. C., Sperling, M. R., & Kahana, M. J. (2019). Hippocampal theta codes for distances in semantic and temporal spaces. *Proceedings of the National Academy of Sciences*, *116*(48), 24343–24352.

Tenenbaum, J. B. (1999). Bayesian modeling of human concept learning. In *Advances in neural information processing systems* (pp. 59–68).

Theves, S., Fernandez, G., & Doeller, C. F. (2019). The hippocampus encodes distances in multidimensional feature space. *Current Biology*, *29*, 1226–1231.

Tolman, E. C. (1948). Cognitive maps in rats and men. *Psychological Review*, *55*, 189–208.

Wu, C. M., Schulz, E., Speekenbrink, M., Nelson, J. D., & Meder, B. (2018). Generalization guides human exploration in vast decision spaces. *Nature Human Behaviour*, *2*, 915—924. doi: 10.1038/s41562-018-0467-4
